# Use Patterns of Leave-on Personal Care Products among Swiss-German Children, Adolescents, and Adults

**DOI:** 10.3390/ijerph10072778

**Published:** 2013-07-03

**Authors:** Eva Manová, Natalie von Goetz, Carmen Keller, Michael Siegrist, Konrad Hungerbühler

**Affiliations:** 1 Institute for Chemical and Bioengineering, ETH Zürich, Wolfgang-Pauli-Strasse 10, 8093, Zürich, Switzerland; E-Mails: eva.manova@chem.ethz.ch (E.M.); konrad.hungerbuehler@chem.ethz.ch (K.H.); 2 Institute for Environmental Decisions, ETH Zürich, Universitätstrasse 16, 8092, Zürich, Switzerland; E-Mails: ckeller@ethz.ch (C.K.); msiegrist@ethz.ch (M.S.)

**Keywords:** personal care products, use prevalence, use frequency, co-use, children

## Abstract

In order to model exposure to ingredients contained in personal care products (PCPs) and assess their potential risks to human health, access to reliable PCP use data, including co-use patterns, is essential. A postal questionnaire survey was conducted to determine the use patterns of eight leave-on PCP categories among the German-speaking population of Switzerland (N = 1,196; ages 0–97 years), providing for the first time in Europe PCP use data for children <12 years of age. The majority of respondents (99%) reported having used at least one of the investigated PCP categories in the past year. Co-use of two or more PCP categories at the same time was common and more complex amongst adults. Regular use of face cream and body lotion was very high in the youngest group of children aged 0–4 years (more than 79% respondents) who may be more vulnerable to certain adverse effects of some PCP ingredients. A comparison with previously collected information on PCP use patterns in Germany and the Netherlands indicates differences in PCP use patterns among European consumers and suggests that surrogate PCP use data from other countries must be used with caution. This work extends the existing knowledge of PCP use patterns and will be useful for new exposure assessments for ingredients contained in PCPs used by the young consumers.

## 1. Introduction

Use of personal care products (PCPs) is an integral part of our daily lives; therefore it is vital to ensure a high level of PCP safety. The various ingredients contained in PCPs have the potential to enter our body via multiple routes, *i.e.*, through dermal absorption, ingestion, and inhalation. There is a debate in the literature as to whether once inside the body PCP ingredients can affect human health. Concerns have been raised about the possible endocrine disrupting activity of numerous PCP ingredients detectable in human matrices, including phthalates [1,2], parabens [3,4], organic ultraviolet (UV) filters [5,6,7,8,9], and triclosan [10]. Furthermore, a variety of nanoparticles, now frequently employed in a wide range of PCPs [11,12] have been shown to have a cytotoxic, genotoxic [13,14,15,16], and neurotoxic potential [17,18]. Many common PCP ingredients may also produce cutaneous side-effects, e.g., contact or photocontact allergies [19,20,21]. 

New ingredients are continuously introduced to the market, creating a need for comprehensive risk research and thorough exposure assessments. Furthermore, a single ingredient is nowadays often contained in different categories of PCPs that can be concurrently used by the same consumer [22]. In order to accurately estimate human exposure and consequently assess the health risk of a PCP ingredient, exposures across the different PCP categories must be aggregated. Realistic PCP use and co-use patterns thus represent a key input to aggregate exposure assessment models. 

Studies collecting use patterns of various PCP categories have been conducted previously in North American [23,24,25,26,27,28] and European populations [29,30,31,32,33]. To date in Europe, however, PCP use patterns among children are very limited and only provided for children aged 12 years and older in the German database VerbraucherAnalyse Jugend (English: Youth Consumer Analysis). Children are believed to be more susceptible to the effects of chemical exposures than adults [34]. They have immature organ systems, higher metabolic rates, and greater body surface area-to-mass ratios. The impact of small changes caused by chemical exposures early in life may not be immediately apparent but may lead to late-life effects [35]. Reliable PCP use data are hence urgently needed in Europe for younger children in order to assess their exposure to various PCP ingredients. Diverse socio-economic, cultural, and environmental factors across Europe may also influence the lifestyles of consumers and affect their PCP use patterns. However, at present, we do not know whether there are appreciable differences in PCP use patterns amongst European consumers since detailed and publicly available national-level data are currently unavailable for most European countries.

The present study was conducted as part of a larger research project focused on organic UV filters in PCPs. It aimed to collect the first set of individual PCP use patterns in the German-speaking population of Switzerland that can be used to calculate consumer exposure to PCPs. Eight widely used leave-on PCP categories were selected (face cream, body lotion, aftershave lotion/balm, hand cream, makeup foundation, lip care, lipstick, sunscreen) for their likelihood of containing organic UV filters. These categories can be used on a regular basis and simultaneously, which has to be taken into account when assessing aggregate exposures; therefore, we also investigated their co-use patterns. For the first time in Europe, we included children below the age of 12 into our study population, to start filling the existing information gap on PCP use in European children. In addition, we sought to compare our results with existing PCP use patterns publicly available in Europe, to provide first insights into the inter-country variation in prevalence and frequency of PCP use, and intra-country variations in PCP use over time. 

## 2. Methods

### 2.1. PCP Use Survey: Settings and Study Design

A postal questionnaire survey (with pre-paid reply envelopes) has been selected as the most feasible and inexpensive methodology for a large-scale study to collect PCP use patterns across all ages as compared to more costly daily diary methodology. The survey comprising open and closed questions was conducted between January and March 2011 to collect PCP use data in the Swiss-German population (approx. 6.5 million people; 66% of the Swiss population [36]). Hereafter, we will use the term Swiss-German to denote the population and the term German-speaking Switzerland if the region is mentioned. Two questionnaire versions were designed: one for children and adolescents and one for adults (English versions of the original German questionnaires can be found in Supplementary Section S1). Throughout this work, we will use the term “children and adolescents” for participants aged 0–17 years (in the discussion further split into “children” aged between 0 and 12 years, and “adolescents” aged between 13 and 17 years), and the term “adults” for those aged ≥18 years. In both questionnaire versions, we asked the household member whose birthday was next to fill out the questionnaire. For respondents under 14 years of age, parents were asked to fill out the questionnaires. General reminder letters were sent four weeks after the initial mail-out. Second reminders, including a new copy of the questionnaire, were sent four weeks later to those who did not respond to the general reminder.

In the self-administered questionnaire, participants were asked to recall their use of the eight investigated PCP categories, namely, face cream, body lotion, aftershave lotion/balm (hereafter referred to as aftershave), hand cream, makeup foundation, lip care, lipstick, and sunscreen, over the past year. We were aware that some of the investigated PCP categories, e.g., lipstick, have traditionally only been used from a certain age on and only by one sex (females). Nevertheless, we asked everyone about the use of all PCP categories to avoid gender/age bias and thus reflect more accurately the actual use patterns in modern societies. 

The core survey questions focused on the prevalence and frequencies of PCP application. For sunscreens, participants were additionally requested to report the product application frequency to different areas of the body, and season of application. The questionnaire also included questions about the specific products used, including the Sun Protection Factor (SPF) values for sunscreens. Participants were asked to provide basic demographic information such as gender, age, and educational attainment (adults only). Data on physical characteristics (body weight, body height, skin type [37]) were also collected. 

### 2.2. Study Population

The questionnaire for children and adolescents was mailed to 1,000 eligible families with children and adolescents aged 0–17 years recruited using a commercially available address database (Schober Addresses-Shop [38]). The adult questionnaire was sent to 2,500 household addresses randomly selected from the Swiss telephone directory. We obtained a response rate of 48.8% for children and adolescents and 36.8% for adults. The response rates were calculated by dividing the number of returned questionnaires by the total number of questionnaires, excluding those sent out to invalid addresses, *i.e.*, for example to respondents who died, moved away or could not answer in German. Our response rates are consistent with previous paper-based postal surveys with two reminders and even slightly higher for children and adolescents [39,40,41]. After exclusion of ineligible, incomplete, and contradictory responses, the final dataset included 397 children and adolescents and 799 adults (exclusion criteria are detailed in the Supplementary Section S2). If a participant chose not to answer specific questions, the responses were coded as missing values. 

### 2.3. Data Analysis

Basic descriptive statistics were used to summarize the demographic and physical characteristics of the study subjects. Prevalence and frequency of PCP use were depicted graphically. Variations in the prevalence of PCP use were analyzed by gender (female, male), age group (four age groups among children and adolescents (0–4, 5–8, 9–12, 13–17); four age groups among adults (18–42, 43–52, 53–65, 66+)), level of education (adults only; primary, secondary school/upper secondary school, university), and skin type (very fair/fair/light brown, medium brown/dark brown/black) using the Chi-squared or Fisher test statistic. The Mann-Whitney U test or the Kruskal–Wallis test were used to assess variations in the frequency of PCP use using the same predictor variables as above. Spearman’s correlation coefficient (R) was used to measure the strength of correlations between frequencies of use of the different PCP categories. Correlations were considered very weak if 0 < R < 0.19, weak if 0.20 < R < 0.39, moderate if 0.40 < R < 0.59, strong if 0.60 < R < 0.79, and very strong if 0.80 < R < 1.00. A p value of <0.05 indicated statistical significance unless noted otherwise. 

Data were analyzed using the statistical software package SPSS 19.0 (SPSS Inc., an IBM company, Chicago, IL, USA, 2010). PCP co-use patterns were generated for the eight PCP categories using an in-house generated Python (version 2.5) script. The complete set of data at an individual level and the Python script details are accessible from the authors upon request.

### 2.4. Inter- and Intra-Country Comparisons of PCP Use Patterns: Data Sources and Treatment

In order to compare the PCP use patterns of consumers in three countries in Western Europe, namely, Switzerland (Swiss-German population), Germany, and the Netherlands, we used survey data on PCP use patterns from two other sources: the German VerbraucherAnalyse (VA) database (more than 31,000 respondents; the second database version for 2011 was used to more closely match the time frame of the other two datasets), and a Dutch dataset (516 respondents) from Biesterbos *et al.* [29]. Several design features of the three datasets required adaptations to ensure a satisfactory level of data comparability. First, the German VA consists of individuals above 12 (VA Jugend, English: Youth) or 14 (VA Klassik, English: Classic) years of age and the Dutch survey consists of individuals above 18 years of age. The VA is not flexible in terms of restricting the dataset to make a population sample of individuals above 18 years; therefore we restricted our Swiss-German population sample to the age range of respondents in the selected version of the VA for the desired PCP category (some PCP categories are not available in the VA Jugend). Also, the Dutch population is restricted by its narrower age-range of respondents (18–70 years). Second, the VA conforms to assumed gender stereotypes (e.g., lipstick use by females only). Therefore, for both the Swiss-German and the Dutch population, we used a reduced dataset that matches the gender of respondents in the VA for a particular PCP category. Third, the VA contains use data for a wide range of facial care product categories for females, e.g., day cream, night cream *etc.* As a result, our “face cream” category for females was excluded from the comparison, as we were unable to match it with a single representative PCP category in the VA. Likewise, “face cream” does not have a matching PCP category for males in the Dutch database. Hence for “face cream”, we only provide a comparison of use between the Swiss-German and the German male populations. Finally, the PCP use frequency categories differ between the three datasets, but we easily merged them into common frequency categories.

Adequate data needed to assess intra-country variations in use of the investigated PCP categories over time are currently lacking for Switzerland. A single study conducted by Berret and colleagues in 2000/2001 is available on the use of sunscreen in families living in Switzerland (N = 1,239; age 0–72 years) [42]. Berret *et al.* [42] focused on sunscreen use during summer holidays, therefore, we compare their findings to our results for sunscreen use (whole-body application) in the summer/autumn season, as we do not have data for summer holidays alone. The focus of the comparison was given to: overall prevalence of sunscreen use, number of daily sunscreen applications, and SPF values of the sunscreen products used.

## 3. Results

### 3.1. Characteristics of the Study Population

Overall, our population sample contained more female (54.6%) than male subjects (45.4%), resulting in a female to male ratio of 1.2:1. For respondents under 14 years of age, mothers completed the questionnaire for the majority of respondents (84.9%). A summary of respondent characteristics (N = 1,196) is shown separately for children and adolescents (combined) and adults in Table 1. Age- and gender-specific distributions of two important exposure factors, namely, body height and body weight (self-reported) of our study population are given in the supplementary Tables S1–S4. 

Self-reported data on Fitzpatrick sun-reactive skin types (see either questionnaire version in Supplementary section S1 for a more detailed description of the different skin types) were available for the vast majority of respondents (N = 1,180; 99%); however, the number of respondents with very fair and dark brown/black skin type was very low. In order to carry out a statistically meaningful data analysis, we merged the Fitzpatrick skin types I, II, and III, that burn easily to moderately, into one category: very fair/fair/light brown. Fitzpatrick skin types IV, and V + VI, which burn minimally to never, were merged into the category medium brown/dark brown/black.

**Table 1:** Summary of respondent characteristics.

CHILDREN AND ADOLESCENTS		
Variable	N	%
**Gender**		
Female (F)	198	49.7
Male (M)	199	50.3
**Age (years)**		
≤4	75	18.9
5–8	103	25.9
9–12	103	25.9
13–17	116	29.2
**Fitzpatrick skin type**		
I	12	3.0
II	78	19.6
III	216	54.4
IV	83	20.9
V + VI	3	0.8
Missing	5	1.3
**CHILDREN** (age 0–12)	**F/mean (SD)**	**M/mean (SD)**
Body height (cm)	121.6 (21.8)	118.9 (20.9)
Body weight (kg)	24.5 (10.2)	22.9 (8.6)
**ADOLESCENTS** (age 13–17)	**F/mean (SD)**	**M/mean (SD)**
Body height (cm)	163.2 (7.3)	165.8 (11.1)
Body weight (kg)	52.0 (7.4)	53.6 (13.5)
**ADULTS**		
**Variable**	**N**	**%**
**Gender**		
F	455	56.9
M	344	43.1
**Age (years)**		
18–42	207	25.9
43–52	203	25.4
53–65	210	26.3
≥66	179	22.4
**Fitzpatrick skin type**		
I (burns immediately, never tans)	15	1.9
II (burns easily, tans slowly with difficulties to brown)	136	17.0
III (burns moderately, tans slowly to brown)	449	56.2
IV (burns rarely, tans fast to moderately brown)	176	22.0
V + VI (almost never burns, tans fast to dark brown)	12	1.5
Missing	11	1.4
**Level of education**		
Primary/secondary school	83	10.4
Upper secondary school	480	60.1
University	227	28.4
Missing	9	1.1
	**F/mean (SD)**	**M/mean (SD)**
Body height (cm)	165.6 (6.7)	177.7 (7.1)
Body weight (kg)	64.3 (10.1)	81.2 (11.0)

### 3.2. PCP Use Patterns

**Figure 1:**
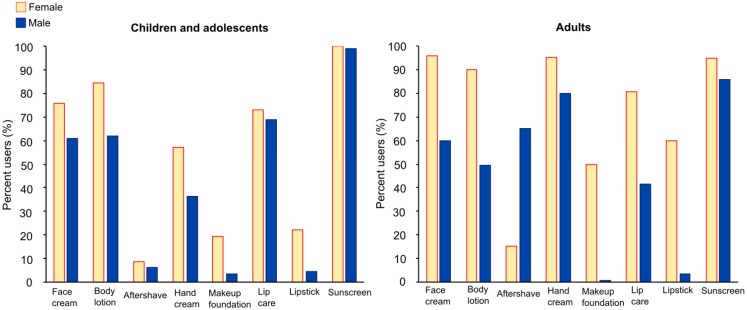
Percentage of PCP users by gender.

**Figure 2:**
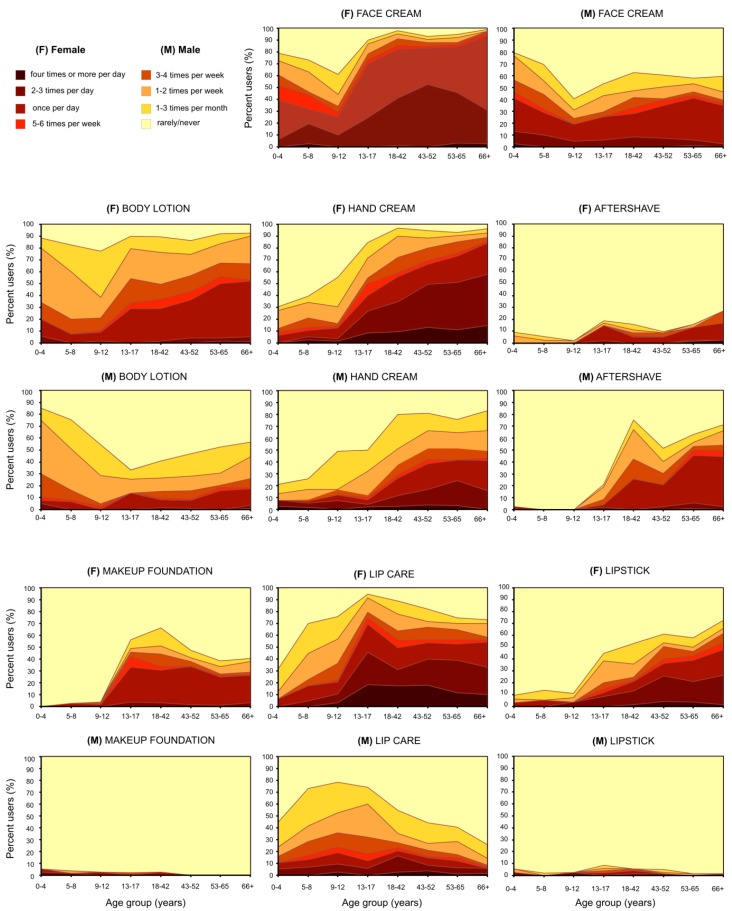
Distributions of use prevalence and frequency of routinely used PCPs across age and by gender.

**Figure 3:**
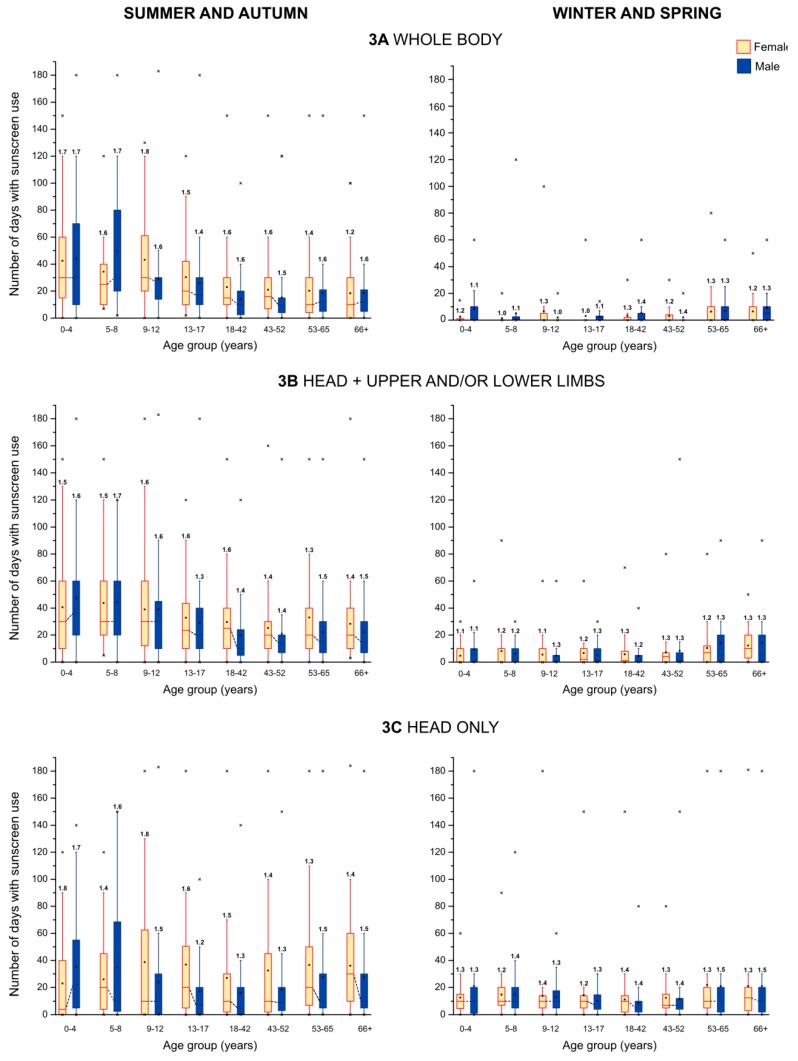
Distributions of frequency of sunscreen use to different body areas across age and gender groups. In each box plot, the lower and upper edges of the box represent the 25th percentile and the 75th percentile, respectively, the square indicates the mean, and the solid horizontal line the median. For better comparison, the medians between genders within each age group are connected by a dotted line. The vertical lines represent the 5th and 95th percentiles of the respective distribution. Outliers are displayed as crosses. The mean (arithmetic) number of sunscreen applications per day is displayed above each box.

While virtually all participants (N = 1,188; 99.3%) in our study reported using at least one of the investigated PCP categories in the past year, the number of respondents using all the categories was low (N = 19; 1.6%). Women used, on average, more PCP categories than men, which was true for both children and adolescents (female, 4; male, 3) and adults (female, 6; male, 4). The overall prevalence of use of the eight PCP categories by gender, and separately for children/adolescents and adults, is shown in Figure 1. Figure 2 depicts how the prevalence and the frequency of PCP use change across age for each gender for the seven PCP categories used routinely throughout the year. Data used for the graphs in Figure 1, Figure 2 are available in the Supplementary Tables S5–S12. The prevalence and the frequency of sunscreen use, including the extent of application of sunscreen to different areas of the body, depend on various types of outdoor activities associated with different seasons. Therefore, in Figure 3 we graphically summarized the frequency of sunscreen application (*i.e.*, the number of days sunscreen was applied and the number of applications per day) by gender, age group, and season. For the purpose of data analysis, however, the frequency of sunscreen use per year was defined as the sum of the number of sunscreen applications to the head for the winter/spring months and to the whole body for the summer/autumn months. In Table 2, we report the prevalence of sunscreen use on different body areas by season (respondents were asked to fill out all response options that applied), and separately for children/adolescents and adults. The prevalence and the frequency of PCP use were compared across gender, age group, education level (adults only), and skin type. The results of these analyses are described below in detail, separately for children/adolescents and adults.

**Table 2:** Prevalence of sunscreen use on different body areas by season (% users).

	C + A (F/M)	AD (F/M)
**Summer and autumn**		
Whole body	95.9 (96.7/95.1)	86.3 (86.6/85.9)
Head, upper and/or lower limbs	94.3 (94.9/93.7)	95.0 (95.6/93.9)
Head only	73.4 (73.0/73.8)	82.1 (83.4/79.9)
**Winter and spring**		
Whole body	26.0 (26.8/25.3)	37.4 (36.8/38.3)
Head, upper and/or lower limbs	49.3 (50.0/48.6)	60.4 (61.4/58.8)
Head only	87.4 (87.2/87.6)	84.6 (85.3/83.6)

C + A (Children and adolescents); AD (Adults); F (Female); M (Male).

#### 3.2.1. PCP Use amongst Children and Adolescents

For children and adolescents the prevalence of use was highest for sunscreen in both females (100.0%) and males (99.0%), followed by use of body lotion for females (84.3%) and lip care for males (68.9%). The prevalence of PCP use was statistically significantly higher for females than males, apart from aftershave, lip care, and sunscreen. With the only exception of sunscreen, the differences in the prevalence of PCP use by age group were statistically significant for all PCP categories. For these seven PCP categories the prevalence of use of face cream and body lotion was highest among 0–4 year olds, whereas for the remaining PCP categories the use was highest for the adolescents (13–17 year olds). It should also be noted that surprisingly aftershave (5.8%) and lipstick (7.2%) were used amongst the youngest children aged 0–4 years. The differences in the prevalence of use by skin type were statistically significant only for sunscreen, with prevalence of use higher in those with very fair/fair/light brown skin compared with those who indicated to have medium brown/dark brown/black skin. Not surprisingly, the body area on which sunscreen was most commonly applied was head in the winter/spring months, and whole body during summer/autumn months, closely followed by the head+upper and/or lower limbs (Table 2). 

Significant gender differences were evident in the frequency of use for six PCP categories (exceptions: aftershave and sunscreen). Statistically significant differences in the frequency of use were also observed for all product categories when analyzed by age group. As seen in Figure 2, for face cream and lip care we observed especially high frequency of use among adolescent females (aged 13–17) as 70% reported using these product categories on a daily basis. 

#### 3.2.2. PCP Use amongst Adults

Amongst adults the prevalence of use was highest for face cream in females (95.9%) and sunscreen in males (85.7%), followed by use of hand cream in both females (95.3%) and males (79.9%). The prevalence of PCP use was statistically significantly higher for females than males, with the only exception of aftershave. Thus the gender differences in the prevalence of PCP use are more pronounced for adults than for children and adolescents. On the other hand, the differences in the prevalence of use by age group were statistically significant for four PCP categories, namely aftershave, makeup foundation, lip care, and sunscreen, and therefore less pronounced for adults than for children and adolescents. For these four PCP categories, the prevalence of use was highest in the youngest population group (18–42), apart from aftershave with the highest prevalence figures observed amongst the oldest age group (66+). Additionally, level of education had an influence on the prevalence of use of body lotion, hand cream, makeup foundation, lipstick, and sunscreen. While the prevalence of use of body lotion and hand cream decreased with increasing education, the prevalence of sunscreen use increased with increasing education. Hence the largest proportion of sunscreen users was found amongst university graduates (92.4%). For decorative cosmetics, *i.e.*, makeup foundation and lipstick, the prevalence of use was highest amongst the respondents with upper secondary school education. Unlike for children and adolescents, the differences in the prevalence of use by skin type were statistically significant for all PCP categories apart from aftershave. The prevalence of use of the seven PCP categories was higher in those with very fair/fair/light brown skin compared with those who indicated to have medium brown/dark brown/black skin. As for children and adolescents, the head-only sunscreen application was more prevalent during winter/spring months, whereas the whole body and the head+upper and/or lower limbs applications of sunscreen were more common for the summer/autumn months. Nevertheless, the prevalence of the whole body sunscreen application was still relatively high in adults (37.4%) even in the winter/spring period.

As for the prevalence of use, there were significant differences in the frequency of PCP use when analyzed by gender and for all eight PCP categories. The mean frequency of use was higher among females for all the PCP categories apart from aftershave, which is used more frequently by males, on average. Signiﬁcant differences in use frequency were found between the four adult age groups for aftershave, makeup foundation, and lip care. There were also significant differences in the frequency of use for face cream, body lotion, makeup foundation, lip care and lipstick, and surprisingly not for sunscreen, when analyzed by skin type. With respect to education level there were significant differences observed in the frequency of use for all PCP categories apart from aftershave. 

#### 3.2.3. Analysis of PCP Co-Use Patterns

As emphasized by previous authors, to calculate the aggregate exposure for individuals and populations it is necessary to have an overall understanding of the key patterns of concurrent application of the different PCP categories [28,43]. We define concurrent use (or co-use) as a regular use of two or more PCP categories. It is worth noting that the PCP selection in this study does not capture all existing PCP categories, which means that it is possible that other PCP categories (leave-on, rinse-off) are co-used with the eight chosen PCP categories. We performed separate analyses for males and females for our two life-stage subgroups (see Table 3 for the five most frequent product combinations and Supplementary Tables S13–S16 for the complete list of all the co-use combinations identified). Our analysis of the PCP co-use patterns shows, similarly to the results of Cowan-Ellsberry and Robison [43], that the actual number of co-use combinations was always smaller than the total number of possible combinations (247). We found 51 and 52 co-use combinations for adult females and males, respectively. For children and adolescents we only identified 34 combinations for females and 24 for males. On the other hand, children and adolescents had higher overall prevalence of concurrent PCP use; 79.8% females and 78.4% males amongst children and adolescents used two or more PCPs simultaneously compared to 64.8% adult females and 71.2% adult males.

**Table 3:** Five most frequent PCP co-use combinations for the eight investigated PCP categories.

Gender	PCP co-use combinations ^a^			
	Children and adolescents	% users	Adults	% users
**F**	SC, FC, BL, HC, LC	18.4	SC, FC, BL, HC, MF, LC, LS	24.1
	SC, FC, BL	10.8	SC, FC, BL, HC, LC	12.5
	SC, FC, BL, LC	10.8	SC, FC, BL, HC, LC, LS	10.5
	SC, FC, BL, HC, MF, LC, LS	6.3	SC, FC, BL, HC, MF, LC	9.2
	SC, BL, LC	5.1	SC, FC, BL, AS, HC, MF, LC, LS	4.7
	SC, BL	4.4		
	SC, FC, BL, HC, MF, LC	4.4		
**M**	SC, FC, BL, LC	21.2	SC, FC, BL, AS, HC, LC	13.5
	SC, FC, BL	11.5	SC, FC, BL, AS, HC	10.6
	SC, LC	10.3	SC, AF, HC	6.5
	SC, FC, BL, HC, LC	10.3	SC, FC, AS, HC, LC	6.1
	SC, BL, LC	8.3	SC, FC, HC	4.9
	SC, HC, LC	6.4		

^a^ Only participants who co-used PCPs and completed the questionnaire fully (*i.e.*, for all eight PCP categories) were considered in the analysis. FC (face cream); BL (body lotion); AS (aftershave); HC (hand cream); MF (makeup foundation); LS (lipstick); LC (lip care); SC (sunscreen).

Overall, our study found very weak to moderate positive correlations (R ranging from 0.12 to 0.58; see Supplementary Figure S1; includes respondents who did not use products) between the use frequencies of different PCP categories. The associations were generally stronger among children and adolescents than among adults and they were also stronger among females than among males. The strongest correlation was found between the use frequencies of hand cream and lip care products for female children and adolescents (R = 0.58, p < 0.01) followed by the correlation between face cream and body lotion for male children and adolescents (R = 0.56, p < 0.01). The latter correlation was in fact the only moderate correlation common for all the subgroups (the remaining common correlations found were very weak to weak). Apart from adult males, the use frequencies of decorative cosmetics, *i.e.*, makeup foundation and lipstick, were also moderately correlated with each other, with the highest correlation coefficient noted for female children and adolescents (R = 0.49, p < 0.01). 

### 3.3. Inter- and Intra-Country Comparison of PCP Use

#### 3.3.1. Comparison of PCP Use Patterns: German-speaking Switzerland, Germany, and the Netherlands

**Figure 4:**
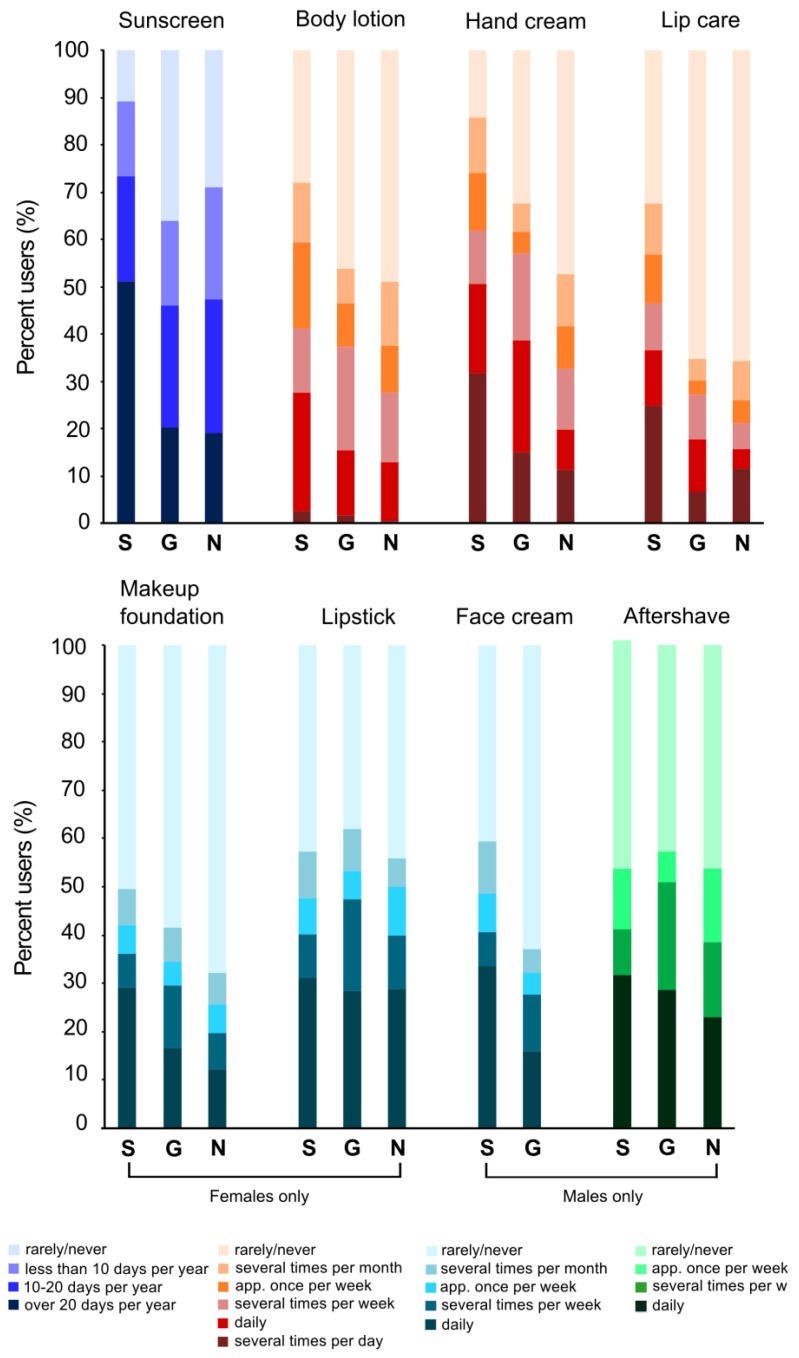
Comparison of PCP use amongst German-speaking Switzerland (S), Germany (G), and the Netherlands (N).

The prevalence of PCP use as well as the distribution of frequencies of use varied amongst German-speaking Switzerland, Germany, and the Netherlands. Overall, the PCP prevalence use rates were highest in German-speaking Switzerland (in six out of the eight PCP categories) and for all PCP categories German-speaking Switzerland had the highest prevalence of the most frequent users. Apart from sunscreen, the lowest prevalence of use was found in the Netherlands. Figure 4 summarizes the PCP use patterns, with non-users (top) to heavy users (bottom). 

#### 3.3.2. Sunscreen Use in Switzerland: Where Do We Stand a Decade Later?

In our study, most respondents (93%) reported sunscreen application on at least one day during the previous summer/autumn period. Similarly, Berret *et al.* reported sunscreen use in 91% participants [42]. Whereas the prevalence of use remained unchanged, the number of daily sunscreen applications has declined over the past decade. In the summer of 2000, the ratio of respondents applying sunscreen once, twice or three and more times per day was 1:1:1.3, in the summer/autumn 2010 it was 1:0.8:0.2 suggesting that the proportion of individuals re-applying sunscreen most frequently has dropped markedly. By contrast, the SPF of sunscreen products used by consumers has increased. In 2000 the ratio of respondents using sunscreens with SPF of <15, 15–20, and >20, was 1:3:4, respectively, whereas a decade later the <15/15–20/>20 ratio was already 1:6:16.

## 4. Discussion

We provide the first assessment of use patterns for eight widely used leave-on PCP categories across all age groups in the Swiss-German population. Our findings build upon and extend the body of current knowledge on PCP use patterns worldwide. Furthermore, data we collected on PCP use amongst young children are an essential prerequisite for exposure assessments of substances contained in PCPs, thus far lacking in the published literature for Europe.

In our female population, 8.8% of female children and adolescents and 15.3% of adult females applied aftershave products in the past year. Although typically perceived as PCPs for males, aftershave products for females exist, in particular those designed for the sensitive bikini area. Our results indicate that aftershave products have become increasingly popular amongst females. Such a finding is not surprising since removal of unwanted body hair is normative in contemporary Western cultures [44,45]. Furthermore, amongst adolescent boys aged 13–17 years, 8.4% used lipstick in the past year. Nowadays, also men use decorative cosmetics, traditionally used by women, to improve their looks. Biesterbos *et al.* have recently reported that 0.5% of adult Dutch males used make-up products (lipstick or lip gloss, makeup foundation, make-up remover, and powder or rouge) [29]. Hence, gender-based stereotyping concerning PCP use becomes less pronounced. Similarly, age-based stereotyping in PCP use tends to diminish. In our study in fact, 56.1% of female adolescent participants aged 13–17 years indicated that they used makeup foundation, previously worn mainly by adult women. Moreover, 7.2% of the youngest children aged 0–4 years used lipstick (see Figure 2). In the US, Wu *et al.* showed that a high percentage of females (45%) age 5 or less used nail polish (not investigated in our study) [28]. On the other hand, our female participants in the oldest age group aged 66 years and older had the highest prevalence of lipstick use (72.6% respondents). As the population ages, older adults also want to retain good appearance in the youth-oriented society and therefore continue using decorative cosmetics until very old age. In order to obtain a realistic representation of people’s actual PCP use patterns that mirrors societal change, it is desirable that future PCP use surveys avoid gender and age product stereotyping.

Knowledge of PCP co-use and the associations of the frequency of use between the individual PCP categories are vital pieces of information for evaluating aggregate exposures of PCP ingredients. Concurrent use of the eight leave-on PCP categories examined in this study was very common, albeit varied between children and adolescents and adults as well as between genders. The overall prevalence rate of concurrent PCP use was higher in children and adolescents than in adults. About 80% of the children and adolescent population (79.8% females; 78.4% males) used at least two PCP categories concurrently on a regular basis in the past year. However, despite the lower prevalence of concurrent use amongst adults (64.8% females; 71.2% males), their co-use patterns were much more complex with a considerably higher number of different PCP combinations (particularly among males) compared to children and adolescents. The substantially greater inter-individual variability in PCP co-use means that it is more difficult to provide an accurate characterization of the aggregate ingredient intake. This finding emphasizes the need for more research focusing on detailed co-use of multiple PCP categories, especially amongst adults (stratified by age and gender and ideally other socio-demographic factors), in order to help to identify the potentially highest exposed subpopulations of consumers. The use frequencies of face cream and body lotion were moderately correlated, the only case that occurred in all age/gender subgroups. The correlation of use frequencies of skincare PCP categories appears to have a similar pattern worldwide: Wu *et al.* found moderately positive correlations between the use frequencies of face moisturizer with body lotion in the US [28]. Similarly, Biesterbos *et al.* found moderately positive correlations between the use frequencies of day/night cream with body lotion in the Netherlands [29]. As highlighted by Wu *et al.* ingredients in topical skin care preparations, which are intended to stay on the skin for prolonged periods of time, can be similar, which may be reflected in individual’s aggregate exposure [28].

Among the three age groups of children, *i.e.*, 0–4 years, 5–8 years, and 9–12 years, the prevalence of use of face cream and body lotion for both genders was highest (>79% of respondents) in the youngest group of children who may be more vulnerable to the potential adverse effects of some PCP ingredients. The specific PCPs that were used on children aged 0–4 years were almost exclusively products labeled for use by infants and children. Parents may assume that infant/children PCPs are rather safe to use; nevertheless, it has been shown previously that even these PCPs may contain potentially harmful ingredients, e.g., fragrances and preservatives [46,47], known to cause allergies and/or are suspected of causing endocrine disruption. Future research should include thorough exploration of use patterns of other PCP categories frequently used by young consumers, including rinse-off products, to accurately assess their aggregate exposure to PCP ingredients.

PCP use patterns might be influenced by regional variations in environmental and socio-economic conditions and also cultural behaviors. Ours is the first comparative analysis of PCP use patterns among three Western European countries: German-speaking Switzerland, Germany, and the Netherlands. Overall, the prevalence and the frequency of PCP use were especially high in German-speaking Switzerland. One possible explanation of the higher use of skin care PCPs, particularly the considerably higher use of sunscreen and lip care, is that many Swiss spend a significant part of their leisure-time outdoors all year round. Switzerland is a largely mountainous country and recreational activities such as hiking, skiing, and rock climbing, are very popular. UV radiation levels are high in mountain areas and reflection from snow further increases exposure to UV radiation. Thus sun protection is highly needed and sunglasses and sunscreen use is encouraged [48]. Additionally, it has been shown previously that in high-altitude ski areas individuals who follow sunscreen advice carefully are also more likely to practice other sun-protection practices, such as the use of UV filter containing lip balm [49]. Also, according to 2010 L’Oreal’s annual report, Switzerland has one of the highest per capita cosmetics consumption in the world, which they assume reflects the high income levels of the country—once basic human needs are satisfied, people are able to spend on luxuries [50]. The higher income levels in Switzerland, compared to Germany and the Netherlands, are likely to enable the Swiss to spend more money on manufactured goods, which in turn may increase their PCP consumption. We acknowledge that our inter-country comparison of PCP use is partially compromised by the differences in survey design of the three studies compared (*i.e.*, different categories of frequency of PCP use, target populations in terms of age and size, and data collection methodologies). Despite these limitations, we are confident that this comparison is meaningful, as it demonstrates, for the first time, that there are differences in PCP use between countries within Europe and that data available from other countries must be used with caution. 

Besides inter-country differences, intra-country variations in the extent and patterns of PCP use might also take place over time within a country. Due to lack of available datasets on PCP use in Switzerland, we were only able to carry out a limited comparison of sunscreen use based on data from Berret *et al.* [42]. It is important to mention here that the annual all-sky sunshine duration (SD) for 2000 was comparable to that of 2010 and in both years the annual all-sky SD was slightly lower than the annual all-sky SD mean [51]. Overall, it seems that the prevalence of sunscreen use in 2000 was very similar to that of 2010. However, compared to a decade ago, our respondents in 2010 used sunscreen products with higher SPF values for more efficient UV protection. At the same time, they appear to assume that fewer applications are adequate for all day exposure. Our results support those of Autier *et al.*, who showed that use of products with high SPF values gives consumers a false sense of security and often leads, counterproductively, to prolonged sun exposure [52]. We have to mention, however, that the decrease in the number of daily applications in our study might have been somewhat influenced by our longer study period (summer and autumn compared to summer only in Berret *et al.* [42]). In the autumn, whole-body sunscreen application is likely to be lower than in the summer. Furthermore, Berret *et al.* also included respondents from the French- and Italian-speaking parts of Switzerland [42].

There are several limitations of the present study that warrant consideration. For children under 14 years of age we relied on one parent/guardian to report the PCP patterns of their child, therefore we might have missed PCP usage unknown to that person. Furthermore, respondents were asked retrospective questions between January and March. Therefore, we assume some level of recall error, especially, in the case of the sunscreen frequency of use during the past year. Also, unlike for PCPs used regularly throughout the year, for the sunscreen frequency of use an open-ended questioning format was used to account for the important change in patterns of sunscreen use between seasons. However, open-ended questions were difficult for respondents to answer and we indeed had to exclude 29 respondents who reported an unrealistically high number of days during which they had used sunscreen (details in the Supplementary Section S2). Hence we further assume some level of error in consumers’ responses because of the questioning format used. On the other hand, we are aware that people who have spent their summer or winter holidays abroad in sunnier regions than Switzerland might have applied sunscreen very frequently. It should also be mentioned that we used open-ended questions to gather information on specific PCPs used by the survey respondents in order to obtain as much details about the products as possible. However, the gathered dataset was extremely complex as the degree of detail given by respondents varied widely. Responses included, e.g., brand names without any further description (e.g., Labello), exact product names (e.g., Labello hydro care), different names describing the same product (e.g., Labello pearl & shine; Labello Perlglanz), and descriptive labels that could be aligned with several products of the same brand (e.g., Labello pure & natural could refer to Labello pure & natural-milk & honey or olive & lemon *etc.*) or even with different brands (e.g., Aloe Vera). Labello, in particular, is also often used as a synonym for lip care in Switzerland and other German-speaking countries. Identification of the individual products to determine the percentages of respondents using the same product is very subjective; therefore we chose not to discuss the PCP brand names that we derived from the questionnaire answers. One also has to bear in mind that the amount of PCP applied on the skin per application influences individual’s exposure levels to ingredients contained in PCPs. However, we have not measured the amount applied for our population sample. In addition, leave-on PCPs investigated can be washed off and the subsequent exposure depends on the time before the next washing. Finally, we cannot rule out that the respondents of our questionnaire might be people who use fewer PCPs/use PCPs less frequently than the average German-Swiss population, as they might be more health-conscious consumers willing to spend the time filling out the questionnaire, unlike their less health-conscious counterparts using more PCPs/PCPs more frequently. 

## 5. Conclusions

Our findings indicate differences in PCP use patterns among European consumers. To address the consumers’ safety risks as best as possible, further efforts are needed to collect internationally comparable and publicly available individual PCP use data across all ages, ideally on an on-going basis using harmonized methodology within Europe as well as worldwide. Such harmonized PCP use data, reflecting national differences, would provide a solid foundation for the estimation of exposure to PCP ingredients. 

## Supplementary Files

Supplementary File 1 Supplementary Information (PDF, 380 KB)

## References

[1] Howdeshell K.L., Wilson V.S., Furr J., Lambright C.R., Rider C.V., Blystone C.R., Hotchkiss A.K., Gray L.E. (2008). A mixture of five phthalate esters inhibits fetal testicular testosterone production in the Sprague-Dawley rat in a cumulative, dose-additive manner. Toxicol. Sci..

[2] Parks L.G., Ostby J.S., Lambright C.R., Abbott B.D., Klinefelter G.R., Barlow N.J., Gray L.E. (2000). The plasticizer diethylhexyl phthalate induces malformations by decreasing fetal testosterone synthesis during sexual differentiation in the male rat. Toxicol. Sci..

[3] Kang K.S., Che J.H., Ryu D.Y., Kim T.W., Li G.X., Lee Y.S. (2002). Decreased sperm number and motile activity on the F1 offspring maternally exposed to butyl p-hydroxybenzoic acid (butyl paraben). J. Vet. Med. Sci..

[4] Oishi S. (2002). Effects of propyl paraben on the male reproductive system. Food Chem. Toxicol..

[5] Inui M., Adachi T., Takenaka S., Inui H., Nakazawa M., Ueda M., Watanabe H., Mori C., Iguchi T., Miyatake K. (2003). Effect of UV screens and preservatives on vitellogenin and choriogenin production in male medaka (*Oryzias latipes*). Toxicology.

[6] Schlumpf M., Cotton B., Conscience M., Haller V., Steinmann B., Lichtensteiger W. (2001). *In vitro* and *in vivo* estrogenicity of UV screens. Environ. Health Perspect..

[7] Schlumpf M., Jarry H., Wuttke W., Ma R., Lichtensteiger W. (2004). Estrogenic activity and estrogen receptor β binding of the UV filter 3-benzylidene camphor. Comparison with 4-methylbenzylidene camphor. Toxicology.

[8] Schlumpf M., Schmid P., Durrer S., Conscience M., Maerkel K., Henseler M., Gruetter M., Herzog I., Reolon S., Ceccatelli R., Faass O., Stutz E., Jarry H., Wuttke W., Lichtensteiger W. (2004). Endocrine activity and developmental toxicity of cosmetic UV filters—An update. Toxicology.

[9] Schmutzler C., Hamann I., Hofmann P.J., Kovacs G., Stemmler L., Mentrup B., Schomburg L., Ambrugger P., Grüters A., Seidlova-Wuttke D., Jarry H., Wuttke W., Köhrle J. (2004). Endocrine active compounds affect thyrotropin and thyroid hormone levels in serum as well as endpoints of thyroid hormone action in liver, heart and kidney. Toxicology.

[10] Foran C.M., Bennett E.R., Benson W.H. (2000). Developmental evaluation of a potential non-steroidal estrogen: Triclosan. Mar. Environ. Res..

[11] Lorenz C., von Goetz N., Scheringer M., Wormuth M., Hungerbühler K. (2011). Potential exposure of German consumers to engineered nanoparticles in cosmetics and personal care products. Nanotoxicology.

[12] Weir A., Westerhoff P., Fabricius L., Hristovski K., von Goetz N. (2012). Titanium dioxide nanoparticles in food and personal care products. Environ. Sci. Technol..

[13] Asare N., Instanes C., Sandberg W.J., Refsnes M., Schwarze P., Kruszewski M., Brunborg G. (2012). Cytotoxic and genotoxic effects of silver nanoparticles in testicular cells. Toxicology.

[14] Jaeger A., Weiss D.G., Jonas L., Kriehuber R. (2012). Oxidative stress-induced cytotoxic and genotoxic effects of nano-sized titanium dioxide particles in human HaCaT keratinocytes. Toxicology.

[15] Kumari M., Khan S.S., Pakrashi S., Mukherjee A., Chandrasekaran N. (2011). Cytogenetic and genotoxic effects of zinc oxide nanoparticles on root cells of Allium cepa. J. Hazard. Mater..

[16] Sharma V., Singh S.K., Anderson D., Tobin D.J., Dhawan A. (2011). Zinc oxide nanoparticle induced genotoxicity in primary human epidermal keratinocytes. J. Nanosci. Nanotechnol..

[17] Long T.C., Saleh N., Tilton R.D., Lowry G.V., Veronesi B. (2006). Titanium dioxide (P25) produces reactive oxygen species in immortalized brain microglia (BV2): Implications for nanoparticle neurotoxicity. Environ. Sci. Technol..

[18] Long T.C., Tajuba J., Sama P., Saleh N., Swartz C., Parker J., Hester S., Lowry G.V., Veronesi B. (2007). Nanosize titanium dioxide stimulates reactive oxygen species in brain microglia and damages neurons *in vitro*. Environ. Health Perspect..

[19] Bryden A.M., Moseley H., Ibbotson S.H., Chowdhury M.M.U., Beck M.H., Bourke J., English J., Farr P., Foulds I.S., Gawkrodger D.J. (2006). Photopatch testing of 1,155 patients: Results of the U.K. multicentre photopatch study group. Br. J. Dermatol..

[20] Kerr A.C., Ferguson J., Haylett A.K., Rhodes L.E., Adamski H., Alomar A., Serra E., Antoniou C., Aubin F., Vigan M. (2012). A European multi-centre photopatch test study (EMCPPTS). Br. J. Dermatol..

[21] Van Oosten E.J., Schuttelaar M.L., Coenraads P.J. (2009). Clinical relevance of positive patch test reactions to the 26 EU-labelled fragrances. Contact Dermatitis.

[22] Dodson R.E., Nishioka M., Standley L.J., Perovich L.J., Brody J.G., Rudel R.A. (2012). Endocrine disruptors and asthma-associated chemicals in consumer products. Environ. Health Perspect..

[23] Final Report of the Personal Care Product Survey. http://www.oeconline.org/our-work/healthier-lives/whats-in-my-makeup-bag/personal-care-product-survey-report.

[24] Loretz L.J., Api A.M., Barraj L.M., Burdick J., Dressler W.E., Gettings S.D., Hsu H.H., Pan Y.H.L., Re T.A., Renskers K.J. (2005). Exposure data for cosmetic products: Lipstick, body lotion, and face cream. Food Chem. Toxicol..

[25] Loretz L., Api A.M., Barraj L., Burdick J., Davis D.A., Dressler W., Gilberti E., Jarrett G., Mann S., Pan Y.H.L. (2006). Exposure data for personal care products: Hairspray, spray perfume, liquid foundation, shampoo, body wash, and solid antiperspirant. Food Chem. Toxicol..

[26] Loretz L.J., Api A.M., Babcock L., Barraj L.M., Burdick J., Cater K.C., Jarrett G., Mann S., Pan Y.H.L., Re T.A., Renskers K.J., Scrafford C.G. (2008). Exposure data for cosmetic products: Facial cleanser, hair conditioner, and eye shadow. Food Chem. Toxicol..

[27] Sathyanarayana S., Karr C.J., Lozano P., Brown E., Calafat A., Liu F., Swan S.H. (2008). Baby care products: Possible sources of infant phthalate exposure. Pediatrics.

[28] Wu X.M., Bennett D.H., Ritz B., Cassady D.L., Lee K., Hertz-Picciotto I. (2010). Usage pattern of personal care products in California households. Food Chem. Toxicol..

[29] Biesterbos J.W.H., Dudzina T., Delmaar C.J.E., Bakker M.I., Russel F.G.M., von Goetz N., Scheepers P.T.J., Roeleveld N. (2013). Usage patterns of personal care products: Important factors for exposure assessment. Food Chem. Toxicol..

[30] Hall B., Tozer S., Safford B., Coroama M., Stelling W., Leneveu-Duchemin M.C., McNamara C., Gibney M. (2007). European consumer exposure to cosmetic products, a framework for conducting population exposure assessments. Food Chem. Toxicol..

[31] Hall B., Steiling W., Safford B., Coroama M., Tozer S., Firmani C., McNamara C., Gibney M. (2011). European consumer exposure to cosmetic products, a framework for conducting population exposure assessments Part 2. Food Chem. Toxicol..

[32] VerbraucherAnalyse [Consumer Analysis] Klassik [Classic] and Jugend [Youth]. http://www.verbraucheranalyse.de/inhalte.

[33] Weegels M.F., van Veen M.P. (2001). Variation of consumer contact with household products: A preliminary investigation. Risk Anal..

[34] Becker M., Edwards S., Massey R.I. (2010). Toxic chemicals in toys and children’s products: Limitations of current responses and recommendations for government and industry. Environ. Sci. Technol..

[35] Grandjean P., Bellinger D., Bergman Å., Cordier S., Davey-Smith G., Eskenazi B., Gee D., Gray K., Hanson M., van den Hazel P. (2008). The Faroes statement: Human health effects of developmental exposure to chemicals in our environment. Basic Clin. Pharmacol. Toxicol..

[36] Swiss Federal Statistical Office (SFSO). http://www.bfs.admin.ch/bfs/portal/de/index/news/04/01.html.

[37] Fitzpatrick T.B. (1988). The validity and practicality of sun reactive skin types I through VI. Arch. Dermatol..

[38] Schober Addresses-Shop. http://www.shop.schober.ch.

[39] Dickson-Spillmann M., Siegrist M., Keller C. (2011). Development and validation of a short, consumer-oriented nutrition knowledge questionnaire. Appetite.

[40] Dickson-Spillmann M., Siegrist M., Keller C., Wormuth M. (2009). Phthalate exposure through food and consumers’ risk perception of chemicals in food. Risk Anal..

[41] Brunner T.A., Siegrist M. (2011). A consumer-oriented segmentation study in the Swiss wine market. Br. Food J..

[42] Berret J., Liardet S., Scaletta C., Panizzon R., Hohlfeld P., Applegate L.A. (2002). Use of sunscreens in families living in Switzerland. Dermatology.

[43] Cowan-Ellsberry C.E., Robison S.H. (2009). Refining aggregate exposure: Example using parabens. Regul. Toxicol. Pharmacol..

[44] Tiggemann M., Kenyon S.J. (1998). The hairlessness norm: The removal of body hair in women. Sex Roles.

[45] Toerien M., Wilkinson S. (2004). Exploring the depilation norm: A qualitative questionnaire study of women’s body hair removal. Qual. Res. Psychol..

[46] Danish Environmental Protection Agency Survey and Health Assessment of Cosmetic Products for Children. http://www2.mst.dk/Udgiv/publications/2007/978-87-7052-638-8/pdf/978-87-7052-639-5.pdf.

[47] Sanchez-Prado L., Alvarez-Rivera G., Lamas J.P., Llompart M., Lores M., Garcia-Jares C. (2013). Content of suspected allergens and preservatives in marketed baby and child care products. Anal. Methods.

[48] World Health Organization Global Solar UV Index: A Practical Guide. http://www.unep.org/pdf/Solar_Index_Guide.pdf.

[49] Buller D.B., Andersen P.A., Walkosz B.J., Scott M.D., Maloy J.A., Dignan M.B., Cutter G.R. (2012). Compliance with sunscreen advice in a survey of adults engaged in outdoor winter recreation at high-elevation ski areas. J. Am. Acad. Dermatol..

[50] L’Oreal Annual Report. http://www.loreal-finance.com/_docs/us/2010-annual-report/LOREAL-2010-AR-volume1DEF.pdf.

[51] Sanchez-Lorenzo A., Wild M. (2012). Decadal variations in estimated surface solar radiation over Switzerland since the late 19th century. Atmos. Chem. Phys..

[52] Autier P., Boniol M., Doré J.F. (2007). Sunscreen use and increased duration of intentional sun exposure: still a burning issue. Int. J. Cancer.

