# Supplementary material for: Use Patterns of Leave-on Personal Care Products among Swiss-German Children, Adolescents, and Adults
Source: Int J Environ Res Public Health. 2013 Jul 3;10(7):2778–98. doi: 10.3390/ijerph10072778 (PMC3734457; doi:10.3390/ijerph10072778)
Supplement: Supplementary File 1 — Supplementary Information (PDF, 380 KB) [file ijerph-10-02778-s001.pdf]

Supplementary Information

Use Patterns of Leave-on Personal Care Products among Swiss-German Children, Adolescents, and Adults

---

Section S1. Questionnaires

Survey on the use of sunscreen in children and adolescents.

This questionnaire focuses on your child. If you have several children, this questionnaire is addressed to the child whose birthday is next and who is younger than 18 years of age. **Please fill out the questionnaire for your child if they are under 14 years of age. If your son/daughter is between the ages of 14 and 17, they are allowed to fill out the questionnaire themselves, with your help if necessary.**

Please keep the following in mind:

- Your responses will be kept completely confidential and anonymous and will be stored by the ETH Zürich (Consumer Behavior group).
- The statistical evaluation will not allow any inference about individuals. We are interested in the attitudes and habits of the Swiss population.
- Responses will be used for scientific non-commercial research and teaching.
- Completing the survey will take around 30 min.

Your questionnaire will be read by an electronic scanning device. Therefore it is very important that you adhere to the following guidelines:

- Please use a black/blue pen (no pencil).
- Please make the cross to fit the size of the box provided.
- Please do not skip any questions and check only one box per question (unless otherwise indicated).
- If you make a mistake, please make a circle around the box and re-enter the correct information.

  By doing this you can, if needed, circle more invalid answers:

  ⊠ (circled) = invalid answer
  ⊠ = valid answer

Please return the completed questionnaire in the enclosed pre-paid envelope by 15 February 2011 to:

ETH Zürich, Consumer Behavior (IED)
Projekt Lebensmittel, CHN (PF 23)
Universitätstrasse 22
CH-8092 Zürich.

Thank you very much for filling out the survey!

Filling out the Questionnaire for Children and Adolescents

The questionnaire should be filled out by the person, who knows best, how often your child used sunscreen in the past year. Adolescents between 14 and 17 years of age are allowed to fill out the questionnaire themselves.

Please indicate who is filling out the questionnaire.

☐ Mother
☐ Father
☐ Adolescent aged between 14 and 17 years of age
☐ Other:

Use of sunscreen

1. Have you used sunscreen on your child at least once during the past year (for adolescents: on yourself)?

☐ Yes (please proceed to question 2)
☐ No (please proceed to question 4)

Frequency of Sunscreen Application

2. In this question we want to find out how often you used sunscreen **on your child (for adolescents: on yourself) in the past year**.

Please indicate **on how many days** (2.1.) and **how frequently** (2.2.) you applied sunscreen to the grey highlighted body areas:

**Body area**	**Summer and autumn**<br>**(May to October)**	**Winter and spring**<br>**(November to April)**
(A) whole-body application<br>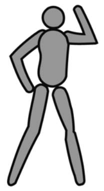	2.1. a total of _____days<br>2.2. on average on one such day<br>☐ 1 time per day<br>☐ 2 times per day<br>☐ 3 times per day<br>☐ 4 or more times per day	2.1. a total of _____days<br>2.2. on average on one such day<br>☐ 1 time per day<br>☐ 2 times per day<br>☐ 3 times per day<br>☐ 4 or more times per day
(B) only head+upper and/or lower limbs<br>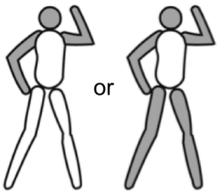	2.1. a total of _____days<br>2.2. on average on one such day<br>☐ 1 time per day<br>☐ 2 times per day<br>☐ 3 times per day<br>☐ 4 or more times per day	2.1. a total of _____days<br>2.2. on average on one such day<br>☐ 1 time per day<br>☐ 2 times per day<br>☐ 3 times per day<br>☐ 4 or more times per day

(C) head-only 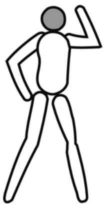	2.1. a total of _____days<br>2.2. on average on one such day<br>☐ 1 time per day<br>☐ 2 times per day<br>☐ 3 times per day<br>☐ 4 or more times per day	2.1. a total of _____days<br>2.2. on average on one such day<br>☐ 1 time per day<br>☐ 2 times per day<br>☐ 3 times per day<br>☐ 4 or more times per day

Name and Sun Protection Factor of the Sunscreens Used

3. Please indicate as specifically and in as much detail as possible the name and the brand (e.g., Nivea Sun Spray; Daylong ultra, Sun Lotion *etc.*) and the sun protection factor (10, 15 *etc.*) of the sunscreen used most frequently on your child (for adolescents: on yourself) during the past summer- and autumn-months.

3.1. In summer and autumn (May to October)

Name and brand of the sunscreen Sun protection factor

Please indicate as specifically and in as much detail as possible the name and the brand (e.g., Nivea Sun Spray; Daylong ultra, Sun Lotion *etc.*) and the sun protection factor (10, 15 *etc.*) of the sunscreen used most frequently **on your child (for adolescents: on yourself)** during the past winter- and spring-months.

3.2. In winter and spring (November to April)

Name and brand of the sunscreen Sun protection factor

3.3. In general, are you using a special sun protection for the lips (e.g., stick or balm) for your child (for adolescents: for yourself)?

☐ Yes. Which one? .... Sun protection factor ....

☐ No.

Frequency of Use of Cosmetics and Toiletries

4. In general, how often did you use the following products for your child (for adolescents: for yourself) during the past year?

	4 or more times per day	2–3 times per day	Once per day	5–6 times per week	3–4 times per week	1–2 times per week	1–3 times per month	Rarely/Never
4.1. Face cream	☐	☐	☐	☐	☐	☐	☐	☐
4.2. Body lotion	☐	☐	☐	☐	☐	☐	☐	☐
4.3. After Shave Balsam/Creme	☐	☐	☐	☐	☐	☐	☐	☐

	4 or more times per day	2–3 times per day	Once per day	5–6 times per week	3–4 times per week	1–2 times per week	1–3 times per month	Rarely/Never
4.4. Hand cream	☐	☐	☐	☐	☐	☐	☐	☐
4.5. Make-up (foundation)	☐	☐	☐	☐	☐	☐	☐	☐
4.6. Lip care	☐	☐	☐	☐	☐	☐	☐	☐
4.7. Lipstick	☐	☐	☐	☐	☐	☐	☐	☐

5. Please indicate for each product category as specifically and in as much detail as possible the name and the brand of the most used product. Please specify for each product, whether you have used it more or less often than every second time.

	Used more than every second time.	Used less than every second time.
5.1. Face cream	☐	☐
5.2. Body lotion	☐	☐
5.3. After Shave Balsam/Creme	☐	☐
5.4. Hand cream	☐	☐
5.5. Make-up (foundation)	☐	☐
5.6. Lip care	☐	☐
5.7. Lipstick	☐	☐

Skin Type of the Child or Adolescent

6. Please indicate which of the following descriptions correspond best to the skin type of your child (for adolescents: of yourself). Skin type 1 is the most sensitive, skin type 6 is the least sensitive. Please check the box of only ONE skin type.

☐	**Celtic type (Skin type 1):**<br>Red or ginger-blond hair,<br>very fair skin (possibly with freckles),<br>burns immediately, never tans.
☐	**Scandinavian type (Skin type 2):**<br>Blond or light brown hair,<br>fair skin,<br>burns easily, the skin tans slowly and with difficulties to brown.
☐	**Mixed type (Skin type 3):**<br>Dark blond or brown hair,<br>light brown skin,<br>burns moderately, tans slowly to brown.

☐	**Mediterranean type (Skin type 4):**<br>Dark brown hair,<br>dark skin,<br>burns rarely, the skin tans fast to moderately brown
☐	**Dark brown and black types (Skin types 5 and 6):**<br>Black hair,<br>very dark skin,<br>almost never burns, the skin tans fast to dark brown.

To Conclude, Please Answer a Few Questions about Your Child (for Adolescents: About Yourself).

7. What is the gender of the child/adolescent?	female			male		
	☐			☐		
8. Which year was the child/adolescent born in?						
9. What is the body weight of the child/adolescent in kilogram?	kg					
10. What is the body height of the child/adolescent in cm?	cm					
11. How many children below the age of 4 live in your household?	No children			1 child	2 children	More than 2 children
	☐			☐	☐	☐
12. How many children between 4 and 20 years of age live in your household?	No children	1 child	2 children	3 child	4 children	More than 4 children
	☐	☐	☐	☐	☐	☐

13. Please indicate on how many days your child has been looked after by others (holidays with a third party care, day care *etc.*) (Days in the kindergarten or at school are not taken into account).

13a. Days in summer and autumn (May to October)
13b. Days in winter and spring (November to April)

Thank you very much for taking the time to fill out the survey!

Survey on the Use of Sunscreen in Adults

Please help us with the within-household random selection of the target respondent. This questionnaire is addressed to the household member whose birthday is next and who is 18 years of age or older.

Thank you very much for taking the time to fill out the survey!

Please keep the following in mind:

- Your responses will be kept completely confidential and anonymous and will be stored by the ETH Zürich (Consumer Behavior group).
- The statistical evaluation will not allow any inference about individuals. We are interested in the attitudes and habits of the Swiss population.
- Responses will be used for scientific non-commercial research and teaching.
- You might have the impression that some questions are similar. This has been done on purpose to increase the accuracy of your answers. Please do not skip any questions.
- Completing the survey will take around 30 min.

Your questionnaire will be read by an electronic scanning device. Therefore it is very important that you adhere to the following guidelines:

- Please use a black/blue pen (no pencil).
- Please make the cross to fit the size of the box provided.
- Please do not skip any questions and check only one box per question (unless otherwise indicated).
- If you make a mistake, please make a circle around the box and re-enter the correct information. By doing this you can, if needed, circle more invalid answers:

(⊠) = invalid answer

⊠ = valid answer

Please return the completed questionnaire in the enclosed pre-paid envelope by 15 February 2011 to:

ETH Zürich, Consumer Behavior (IED)
Projekt Lebensmittel, CHN (PF 23)
Universitätstrasse 22
CH-8092 Zürich.

Thank you very much for filling out the survey!

Use of Sunscreen

1. Have you used sunscreen at least once during the past year?

☐ Yes (please proceed to question 2)

☐ No (please proceed to question 4)

Frequency of Sunscreen Application

2. In this question we want to find out how often you used sunscreen in the past year.

Please indicate **on how many days** (2.1) and **how frequently** (2.2) you applied sunscreen to the grey highlighted body areas:

**Body area**	**Summer and autumn**<br>**(May to October)**	**Winter and spring**<br>**(November to April)**
(A) whole-body application 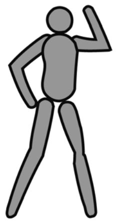	2.1. a total of _____days<br>2.2. on average on one such day<br>☐ 1 time per day<br>☐ 2 times per day<br>☐ 3 times per day<br>☐ 4 or more times per day	2.1. a total of _____days<br>2.2. on average on one such day<br>☐ 1 time per day<br>☐ 2 times per day<br>☐ 3 times per day<br>☐ 4 or more times per day
(B) only head+upper and/or lower limbs 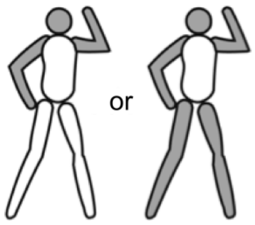	2.1 a total of _____days<br>2.2 on average on one such day<br>☐ 1 time per day<br>☐ 2 times per day<br>☐ 3 times per day<br>☐ 4 or more times per day	2.1 a total of _____days<br>2.2 on average on one such day<br>☐ 1 time per day<br>☐ 2 times per day<br>☐ 3 times per day<br>☐ 4 or more times per day
(C) head-only 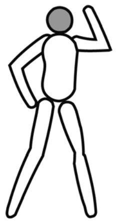	2.1 a total of _____days<br>2.2 on average on one such day<br>☐ 1 time per day<br>☐ 2 times per day<br>☐ 3 times per day<br>☐ 4 or more times per day	2.1 a total of _____days<br>2.2 on average on one such day<br>☐ 1 time per day<br>☐ 2 times per day<br>☐ 3 times per day<br>☐ 4 or more times per day

Name and Sun Protection Factor of the Sunscreens Used

3. Please indicate as specifically and in as much detail as possible the name and the brand (e.g., Nivea Sun Spray; Daylong ultra, Sun Lotion *etc.*) and the sun protection factor (10, 15 *etc.*) of the sunscreen you used most frequently during the past summer- and autumn-months.

3.1. In summer and autumn (May to October)

Name and brand of the sunscreen Sun protection factor

Please indicate as specifically and in as much detail as possible the name and the brand (e.g., Nivea Sun Spray; Daylong ultra, Sun Lotion *etc.*) and the sun protection factor (10, 15 *etc.*) of the sunscreen you used most frequently during the past winter- and spring-months.

3.2. In winter and spring (November to April)

Name and brand of the sunscreen Sun protection factor

3.3. In general, are you using a special sun protection for the lips (e.g., stick or balm)?

☐ Yes. Which one? .... Sun protection factor ....

☐ No.

Frequency of Use of Cosmetics And Toiletries

4. In general, how often did you use the following products during the past year?

	4 or more times per day	2–3 times per day	Once per day	5–6 times per week	3–4 times per week	1–2 times per week	1–3 times per month	Rarely/Never
4.1. Face cream	☐	☐	☐	☐	☐	☐	☐	☐
4.2. Body lotion	☐	☐	☐	☐	☐	☐	☐	☐
4.3. After Shave Balsam/Creme	☐	☐	☐	☐	☐	☐	☐	☐
4.4. Hand cream	☐	☐	☐	☐	☐	☐	☐	☐
4.5. Make-up (foundation)	☐	☐	☐	☐	☐	☐	☐	☐
4.6. Lip care	☐	☐	☐	☐	☐	☐	☐	☐
4.7. Lipstick	☐	☐	☐	☐	☐	☐	☐	☐

5. Please indicate for each product category as specifically and in as much detail as possible the name and the brand of the most used product. Please specify for each product, whether you have used it more or less often than every second time.

	Used more than every second time.	Used less than every second time.
5.1. Face cream	☐	☐
5.2. Body lotion	☐	☐

5.3. After Shave Balsam/Creme	☐	☐
5.4. Hand cream	☐	☐
5.5. Make-up (foundation)	☐	☐
5.6. Lip care	☐	☐
5.7. Lipstick	☐	☐

Your Skin Type

6. Please indicate which of the following descriptions correspond best to your skin type. Skin type 1 is the most sensitive, skin type 6 is the least sensitive. Please check the box of only ONE skin type.

☐	**Celtic type (Skin type 1):**<br>Red or ginger-blond hair,<br>very fair skin (possibly with freckles),<br>burns immediately, never tans.
☐	**Scandinavian type (Skin type 2):**<br>Blond or light brown hair,<br>fair skin,<br>burns easily, the skin tans slowly and with difficulties to brown.
☐	**Mixed type (Skin type 3):**<br>Dark blond or brown hair,<br>light brown skin,<br>burns moderately, tans slowly to brown.
☐	**Mediterranean type (Skin type 4):**<br>Dark brown hair,<br>dark skin,<br>burns rarely, the skin tans fast to moderately brown
☐	**Dark brown and black types (Skin types 5 and 6):**<br>Black hair,<br>very dark skin,<br>almost never burns, the skin tans fast to dark brown.

Your Attitude

7. Please indicate how much you agree with each statement.

	Do not agree at all				Agree completely	
	1	2	3	4	5	6
7.1. I am very concerned about my health.	☐	☐	☐	☐	☐	☐
7.2. In general, I pay attention to my inner feelings regarding my health.	☐	☐	☐	☐	☐	☐

	Do not agree at all				Agree completely	
	1	2	3	4	5	6
7.3. I always take care of my health.	☐	☐	☐	☐	☐	☐
7.4. I usually know, what my current state of health is.	☐	☐	☐	☐	☐	☐
7.5. I am vigilant of changes in my health.	☐	☐	☐	☐	☐	☐
7.6. My health is very important to me.	☐	☐	☐	☐	☐	☐

8. In the following, you will find statements about synthetic substances. Please indicate how much you agree with each statement. Synthetic substances are artificially (chemically) produced substances.

	Do not agree at all				Agree completely	
	1	2	3	4	5	6
8.1. The use of synthetic substances has rather improved than harmed our health.	☐	☐	☐	☐	☐	☐
8.2. I am not concerned about small amounts of synthetic substances in our food and groundwater.	☐	☐	☐	☐	☐	☐
8.3. People are unnecessarily concerned with the potential negative effects of synthetic substances on our health.	☐	☐	☐	☐	☐	☐
8.4. Synthetic substances play an important role in the advancement of the society.	☐	☐	☐	☐	☐	☐
8.5. Our society has to deal with more significant problems than the risks of synthetic substances.	☐	☐	☐	☐	☐	☐
8.6. Synthetic substances and the potential risks associated with them make me worried.	☐	☐	☐	☐	☐	☐
8.7. No expenses should be spared to decrease risks from synthetic substances.	☐	☐	☐	☐	☐	☐
8.8. I do everything I can to avoid contact with synthetic substances in my daily life.	☐	☐	☐	☐	☐	☐
8.9. The exposure to synthetic substances should always be risk-free.	☐	☐	☐	☐	☐	☐
8.10. I would prefer to live in a world where synthetic substances do not exist.	☐	☐	☐	☐	☐	☐

9. In the following, you will find general statements about the benefits of sunrays. Please indicate your assessment.

How much do you appreciate the benefit of sun rays for …	Not large at all				Very large	
	1	2	3	4	5	6
9.1. the production of vitamin D in the skin?	☐	☐	☐	☐	☐	☐
9.2. the prevention of diseases?	☐	☐	☐	☐	☐	☐

10. In the following, you will find general statements about the risks of sunrays. Please indicate your assessment.

How high do you think is the risk from sunrays for the development of …	Not large at all				Very large	
	1	2	3	4	5	6
10.1. premature wrinkles and skin aging?	☐	☐	☐	☐	☐	☐
10.2. skin spots?	☐	☐	☐	☐	☐	☐
10.3. skin cancer?	☐	☐	☐	☐	☐	☐

To Conclude, Please Answer a Few Questions about Yourself.

<table>
<tr><td rowspan="2">11. What is your gender?</td><td colspan="3">female</td><td colspan="3">male</td></tr>
<tr><td colspan="3">☐</td><td colspan="3">☐</td></tr>
<tr><td>12. Which year were you born in?</td><td colspan="6"></td></tr>
<tr><td>13. What is your body weight in kilogram?</td><td colspan="6">kg</td></tr>
<tr><td>14. What is your body height in cm?</td><td colspan="6">cm</td></tr>
<tr><td rowspan="2">15. How many children below the age of 4 live in your household?</td><td colspan="3">No children</td><td>1 child</td><td>2 children</td><td>More than 2 children</td></tr>
<tr><td colspan="3">☐</td><td>☐</td><td>☐</td><td>☐</td></tr>
<tr><td rowspan="2">16. How many children between 4 and 20 years of age live in your household?</td><td>No children</td><td>1 child</td><td>2 children</td><td>3 child</td><td>4 children</td><td>More than 4 children</td></tr>
<tr><td>☐</td><td>☐</td><td>☐</td><td>☐</td><td>☐</td><td>☐</td></tr>
</table>

17. Please indicate the highest level of education successfully completed

Primary school	☐
Secondary school	☐
Upper secondary: Vocational	☐
Upper secondary: General	☐
University	☐

Thank you very much for taking the time to fill out the survey!

Section S2. Study Population: Restriction Criteria

From the population sample of children and adolescents (N = 464) we excluded 18 respondents with missing age details and 11 respondents older than 17 years to ensure that all respondents belonged to our intended target population aged 0–17 years. A total of 6 respondents was excluded because they did not report their gender. Cases with missing or biologically unrealistic data on body height and weight were also deleted (N = 20). A total of 7 respondents did not indicate who filled out the questionnaire and 3 respondents were identified as not eligible to complete the questionnaire (e.g., children under 14 years of age who answered the questionnaire themselves) and hence their answers deleted. Finally, 2 respondents were deleted due to unrealistic data in sunscreen usage in one season. The number of days by season was: summer/autumn (May to October—184 days); winter/spring (November to April—181 days, with February having 28 days). Thus, the final study cohort of children and adolescents included 397 respondents. Similarly, from the population sample of adults (N = 864), we excluded 19 respondents with missing age and gender details, respectively. Cases with missing or biologically unrealistic data on body height and weight were also deleted (N = 11). We also excluded 5 respondents who returned empty/very incomplete questionnaires and 2 duplicate respondents. Further, 1 respondent was excluded as he/she indicated no sunscreen use in the past year but still answered questions on sunscreen use. Finally, 27 cases were deleted due to unrealistic data in sunscreen usage in one season (same criteria applied as discussed above for children). The remaining cohort of adults included 799 respondents.

Section S3. Supplementary Tables and Figures

Table S1. Mean and percentile body weights (kg) for females.

Age group (years)	N	Mean (kg)	SD	Min (kg)	10th (kg)	25th (kg)	Median (kg)	75th (kg)	90th (kg)	Max (kg)
≤4	35	13.3	3.0	8.0	8.6	11.0	14.0	15.0	17.4	20.0
5–8	42	21.6	5.4	13.0	15.3	17.0	21.0	24.0	30.0	40.0
9–12	59	33.1	7.8	20.0	23.0	28.0	32.0	39.0	45.0	50.0
13–17	62	52.0	7.4	32.0	40.0	48.8	53.0	58.0	60.0	65.0
18–42	131	65.4	11.5	42.0	54.2	58.0	63.0	70.0	80.0	115.0
43–52	120	63.7	9.3	41.0	54.1	58.0	63.0	68.0	76.9	95.0
53–65	117	63.2	9.6	47.0	51.6	57.0	62.0	70.0	75.0	95.0
≥66	87	64.8	9.5	48.0	54.0	58.0	64.0	70.0	77.4	95.0

Table S2. Mean and percentile body weights (kg) for males.

Age group (years)	N	Mean (kg)	SD	Min (kg)	10th (kg)	25th (kg)	Median (kg)	75th (kg)	90th (kg)	Max (kg)
≤4	40	14.1	2.9	10.0	10.1	11.3	14.0	16.0	18.0	22.0
5–8	61	21.2	4.0	15.0	16.0	18.0	21.0	24.0	27.8	30.0
9–12	44	33.2	5.7	20.0	26.5	29.3	32.5	36.5	41.0	48.0
13–17	54	53.6	13.5	35.0	38.0	41.0	50.0	64.3	74.0	82.0
18–42	76	81.5	10.7	60.0	69.7	74.0	80.0	88.0	96.5	115.0
43–52	83	83.4	12.8	61.0	68.4	74.0	82.0	90.0	100.0	125.0
53–65	93	81.0	9.6	60.0	70.0	75.0	80.0	85.5	95.0	111.0
≥66	92	79.0	10.5	56.0	66.0	71.0	80.0	84.0	93.8	110.0

Table S3. Mean and percentile body heights (cm) for females.

Age group (years)	N	Mean (cm)	SD	Min (cm)	10th (cm)	25th (cm)	Median (cm)	75th (cm)	90th (cm)	Max (cm)
≤4	35	92.9	11.3	70.0	74.6	83.0	97.0	102.0	104.4	109.0
5–8	42	119.1	10.1	98.0	105.3	110.0	120.0	125.8	131.4	142.0
9–12	59	140.3	10.2	123.0	128.0	130.0	140.0	150.0	153.0	165.0
13–17	62	163.2	7.4	142.0	155.0	160.0	163.0	167.3	173.0	179.0
18–42	131	167.6	6.8	152.0	159.2	163.0	168.0	172.0	175.0	198.0
43–52	120	166.4	6.4	150.0	158.0	162.0	166.0	171.8	175.0	180.0
53–65	117	165.2	6.7	150.0	157.0	160.0	165.0	170.0	174.0	184.0
≥66	87	162.0	5.8	148.0	153.0	158.0	162.0	166.0	168.0	176.0

Table S4. Mean and percentile body heights (cm) for males.

Age group (years)	N	Mean (cm)	SD	Min (cm)	10th (cm)	25th (cm)	Median (cm)	75th (cm)	90th (cm)	Max (cm)
≤4	40	94.4	11.4	72.0	80.1	83.8	98.0	103.8	109.8	120.0
5–8	61	118.0	10.3	98.0	105.0	110.0	118.0	125.0	130.8	142.0
9–12	44	142.4	8.5	125.0	130.0	137.0	141.5	148.0	153.0	165.0
13–17	54	165.8	11.1	145.0	150.5	155.8	166.0	175.0	182.0	185.0
18–42	76	179.3	5.9	164.0	172.0	176.0	179.5	183.8	186.3	194.0
43–52	83	180.8	7.0	164.0	170.4	176.0	180.0	185.0	190.0	198.0
53–65	93	177.1	6.8	164.0	168.4	172.0	176.0	182.0	186.6	193.0
≥66	92	174.2	7.0	154.0	166.3	170.0	174.0	180.0	183.7	190.0

Table S5. Prevalence of PCP use in all respondents, as well as female and male respondents, for the eight investigated PCP categories.

**Product**	**N respondents**	**N users**	**% users**	**N female respondents**	**N female users**	**% female users**	**N male respondents**	**N male users**	**% male users**
**Children and adolescents**									
Face cream	384	262	68.2	193	146	75.6	191	116	60.7
Body lotion	385	281	73.0	191	161	84.3	194	120	61.9
Aftershave	349	26	7.4	170	15	8.8	179	11	6.1
Hand cream	374	175	46.8	187	107	57.2	187	68	36.4
Makeup foundation	363	41	11.3	182	35	19.2	181	6	3.3
Lip care	379	269	71.0	189	138	73.0	190	131	68.9
Lipstick	365	49	13.4	184	41	22.3	181	8	4.4
Sunscreen	397	395	99.5	198	198	100.0	199	197	99.0
**Adults**									
**Product**	**N respondents**	**N users**	**% users**	**N female respondents**	**N female users**	**% female users**	**N male respondents**	**N male users**	**% male users**
Face cream	769	621	80.8	444	426	95.9	325	195	60.0
Body lotion	765	558	72.9	444	399	89.9	321	159	49.5
Aftershave	654	263	40.2	326	50	15.3	328	213	64.9
Hand cream	780	692	88.7	447	426	95.3	333	266	79.9
Makeup foundation	705	209	29.6	416	207	49.8	289	2	0.7
Lip care	728	468	64.3	423	341	80.6	305	127	41.6
Lipstick	714	265	37.1	426	255	59.9	288	10	3.5
Sunscreen	776	705	90.9	440	417	94.8	336	288	85.7

Table S6. Prevalence and frequency of face cream use in females and males by age group.

Application frequency	0–4 years	5–8 years	9–12 years	13–17 years	18–42 years	43–52 years	53–65 years	66+ years
**Females**								
Four times or more per day	0.0	2.4	0.0	0.0	0.8	0.0	3.4	2.5
2–3 times per day	6.1	17.1	10.2	25.0	40.0	52.5	42.2	28.8
Once per day	33.3	12.2	15.3	45.0	41.5	30.5	38.8	62.5
5–6 times per week	12.1	12.2	3.4	3.3	3.1	1.7	1.7	2.5
3–4 times per week	9.1	2.4	5.1	5.0	5.4	3.4	1.7	1.3
1–2 times per week	12.1	17.1	10.2	8.3	4.6	2.5	4.3	0.0
1–3 times per month	6.1	9.8	16.9	3.3	2.3	2.5	2.6	1.3
Rarely/Never	21.2	26.8	39.0	10.0	2.3	6.8	5.2	1.3
**Males**								
Application frequency	0–4 years	5–8 years	9–12 years	13–17 years	18–42 years	43–52 years	53–65 years	66+ years
Four times or more per day	2.6	0.0	0.0	0.0	0.0	0.0	1.1	0.0
2–3 times per day	10.3	10.2	4.8	5.9	8.3	7.4	4.5	2.4
Once per day	28.2	18.6	14.3	19.6	19.4	27.2	34.8	32.5
5–6 times per week	5.1	3.4	0.0	0.0	5.6	4.9	2.2	2.4
3–4 times per week	10.3	11.9	4.8	3.9	8.3	1.2	3.4	2.4
1–2 times per week	20.5	11.9	7.1	13.7	5.6	9.9	6.7	7.2
1–3 times per month	2.6	13.6	9.5	9.8	15.3	9.9	4.5	13.3
Rarely/Never	20.5	30.5	59.5	47.1	37.5	39.5	42.7	39.8

Table S7. Prevalence and frequency of body lotion use in females and males by age group.

Application frequency	0–4 years	5–8 years	9–12 years	13–17 years	18–42 years	43–52 years	53–65 years	66+ years
**Females**								
Four times or more per day	0.0	0.0	0.0	0.0	0.0	0.0	1.7	1.3
2–3 times per day	5.7	0.0	1.8	0.0	1.5	4.2	2.6	3.8
Once per day	14.3	7.5	7.0	28.8	27.5	32.2	45.7	46.8
5–6 times per week	0.0	0.0	1.8	5.1	7.6	6.8	6.0	1.3
3–4 times per week	14.3	12.5	10.5	20.3	13.0	13.6	11.2	13.9
1–2 times per week	45.7	40.0	17.5	25.4	26.7	17.8	16.4	22.8
1–3 times per month	8.6	22.5	38.6	10.2	13.0	11.9	8.6	2.5
Rarely/Never	11.4	17.5	22.8	10.2	10.7	13.6	7.8	7.6

Table S7. *Cont.*

**Males**								
Application frequency	0–4 years	5–8 years	9–12 years	13–17 years	18–42 years	43–52 years	53–65 years	66+ years
Four times or more per day	0.0	0.0	0.0	0.0	0.0	0.0	0.0	1.3
2–3 times per day	5.0	0.0	0.0	0.0	1.4	1.2	0.0	2.5
Once per day	2.5	6.6	0.0	13.7	7.0	6.2	15.7	13.8
5–6 times per week	2.5	1.6	2.4	0.0	1.4	1.2	2.2	1.3
3–4 times per week	20.0	8.2	2.4	0.0	5.6	7.4	2.2	7.5
1–2 times per week	45.0	34.4	23.8	11.8	11.3	12.3	10.1	18.8
1–3 times per month	10.0	24.6	26.2	7.8	14.1	18.5	21.3	12.5
Rarely/Never	15.0	24.6	45.2	66.7	59.2	53.1	48.3	42.5

Table S8. Prevalence and frequency of aftershave use in females and males by age group.

**Females**								
Application frequency	0–4 years	5–8 years	9–12 years	13–17 years	18–42 years	43–52 years	53–65 years	66+ years
Four times or more per day	0.0	0.0	0.0	0.0	0.0	0.0	1.3	2.4
2–3 times per day	0.0	0.0	0.0	2.1	0.0	0.0	1.3	0.0
Once per day	0.0	0.0	0.0	12.5	5.3	5.3	10.5	14.6
5–6 times per week	0.0	0.0	0.0	0.0	0.9	0.0	0.0	0.0
3–4 times per week	0.0	0.0	0.0	0.0	2.6	3.2	0.0	9.8
1–2 times per week	6.3	2.8	1.9	2.1	2.6	0.0	2.6	0.0
1–3 times per month	3.1	2.8	0.0	2.1	4.4	1.1	0.0	0.0
Rarely/Never	90.6	94.4	98.1	81.3	84.2	90.5	84.2	73.2
**Males**								
Application frequency	0–4 years	5–8 years	9–12 years	13–17 years	18–42 years	43–52 years	53–65 years	66+ years
Four times or more per day	0.0	0.0	0.0	0.0	0.0	0.0	1.1	0.0
2–3 times per day	0.0	0.0	0.0	2.1	0.0	2.4	4.4	2.4
Once per day	2.7	0.0	0.0	2.1	26.0	18.3	39.6	42.7
5–6 times per week	0.0	0.0	0.0	0.0	0.0	0.0	4.4	4.9
3–4 times per week	0.0	0.0	0.0	4.3	16.4	9.8	3.3	4.9
1–2 times per week	0.0	0.0	0.0	10.6	24.7	9.8	3.3	12.2
1–3 times per month	0.0	0.0	0.0	2.1	8.2	11.0	7.7	3.7
Rarely/Never	97.3	100.0	100.0	78.7	24.7	48.8	36.3	29.3

Table S9. Prevalence and frequency of hand cream use in females and males by age group.

**Females**								
Application frequency	0–4 years	5–8 years	9–12 years	13–17 years	18–42 years	43–52 years	53–65 years	66+ years
Four times or more per day	0.0	2.6	1.8	8.3	9.3	13.4	11.2	14.5
2–3 times per day	0.0	2.6	1.8	18.3	25.6	36.1	39.7	43.4
Once per day	6.1	5.3	8.9	13.3	20.9	16.8	22.4	26.5
5–6 times per week	3.0	2.6	1.8	10.0	3.1	3.4	2.6	1.2
3–4 times per week	3.0	7.9	1.8	5.0	13.2	10.1	9.5	3.6
1–2 times per week	15.2	13.2	14.3	16.7	17.8	8.4	5.2	3.6
1–3 times per month	3.0	5.3	25.0	13.3	7.0	6.7	2.6	3.6
Rarely/Never	69.7	60.5	44.6	15.0	3.1	5.0	6.9	3.6
**Males**								
Application frequency	0–4 years	5–8 years	9–12 years	13–17 years	18–42 years	43–52 years	53–65 years	66+ years
Four times or more per day	2.6	1.7	0.0	2.0	2.6	3.6	3.3	0.0
2–3 times per day	5.3	3.4	7.3	2.0	9.2	13.3	20.7	15.9
Once per day	0.0	1.7	4.9	4.0	14.5	21.7	17.4	25.6
5–6 times per week	0.0	0.0	0.0	2.0	2.6	3.6	0.0	2.4
3–4 times per week	0.0	1.7	4.9	2.0	9.2	9.6	9.8	6.1
1–2 times per week	5.3	8.6	0.0	20.0	13.2	14.5	13.0	17.1
1–3 times per month	7.9	8.6	31.7	18.0	28.9	14.5	10.9	17.1
Rarely/Never	78.9	74.1	51.2	50.0	19.7	19.3	25.0	15.9

Table S10. Prevalence and frequency of makeup foundation use in females and males by age group.

**Females**								
Application frequency	0–4 years	5–8 years	9–12 years	13–17 years	18–42 years	43–52 years	53–65 years	66+ years
Four times or more per day	0.0	0.0	0.0	0.0	0.0	0.0	0.0	0.0
2–3 times per day	0.0	0.0	0.0	3.5	3.2	1.7	0.9	2.9
Once per day	0.0	2.7	1.8	29.8	27.2	31.9	23.6	23.2
5–6 times per week	0.0	0.0	0.0	8.8	3.2	0.0	1.9	0.0
3–4 times per week	0.0	0.0	1.8	3.5	10.4	4.3	0.9	2.9
1–2 times per week	0.0	0.0	0.0	3.5	7.2	3.4	6.6	8.7
1–3 times per month	0.0	0.0	0.0	7.0	15.2	6.0	4.7	2.9
Rarely/Never	100.0	97.3	96.4	43.9	33.6	52.6	61.3	59.4

Table S10. *Cont.*

**Males**								
Application frequency	0–4 years	5–8 years	9–12 years	13–17 years	18–42 years	43–52 years	53–65 years	66+ years
Four times or more per day	0.0	0.0	0.0	0.0	0.0	0.0	0.0	0.0
2–3 times per day	2.7	1.8	0.0	0.0	0.0	0.0	0.0	0.0
Once per day	2.7	0.0	2.4	0.0	2.8	0.0	0.0	0.0
5–6 times per week	0.0	0.0	0.0	0.0	0.0	0.0	0.0	0.0
3–4 times per week	0.0	0.0	0.0	2.1	0.0	0.0	0.0	0.0
1–2 times per week	0.0	1.8	0.0	0.0	0.0	0.0	0.0	0.0
1–3 times per month	0.0	0.0	0.0	0.0	0.0	0.0	0.0	0.0
Rarely/Never	94.6	96.4	97.6	97.9	97.2	100	100	100.0

Table S11. Prevalence and frequency of lip care use in females and males by age group.

**Females**								
Application frequency	0–4 years	5–8 years	9–12 years	13–17 years	18–42 years	43–52 years	53–65 years	66+ years
Four times or more per day	0.0	0.0	3.4	18.6	17.5	17.9	11.8	10.0
2–3 times per day	0.0	5.0	6.9	27.1	13.5	22.2	27.3	22.9
Once per day	6.3	12.5	10.3	23.7	18.3	13.7	13.6	21.4
5–6 times per week	0.0	0.0	1.7	5.1	6.3	2.6	3.6	1.4
3–4 times per week	0.0	5.0	13.8	5.1	7.9	10.3	8.2	2.9
1–2 times per week	6.3	22.5	20.7	11.9	14.3	5.1	5.5	11.4
1–3 times per month	18.8	25.0	19.0	3.4	11.1	10.3	4.5	2.9
Rarely/Never	68.8	30.0	24.1	5.1	11.1	17.9	25.5	27.1
**Males**								
Application frequency	0–4 years	5–8 years	9–12 years	13–17 years	18–42 years	43–52 years	53–65 years	66+ years
Four times or more per day	0.0	0.0	2.4	0.0	2.7	3.7	1.2	1.4
2–3 times per day	5.3	6.7	7.1	6.0	13.7	4.9	4.9	4.3
Once per day	5.3	6.7	9.5	6.0	4.1	6.2	6.1	0.0
5–6 times per week	0.0	3.3	4.8	6.0	2.7	1.2	2.4	1.4
3–4 times per week	5.3	11.7	11.9	14.0	4.1	4.9	2.4	1.4
1–2 times per week	7.9	13.3	16.7	28.0	8.2	6.2	11.0	5.8
1–3 times per month	21.1	31.7	26.2	14.0	19.2	17.3	12.2	11.6
Rarely/Never	55.3	26.7	21.4	26.0	45.2	55.6	59.8	73.9

Table S12. Prevalence and frequency of lipstick use in females and males by age group.

**Females** Application frequency	0–4 years	5–8 years	9–12 years	13–17 years	18–42 years	43–52 years	53–65 years	66+ years
Four times or more per day	0.0	0.0	0.0	0.0	1.6	4.3	3.6	1.4
2–3 times per day	0.0	0.0	3.6	8.3	11.7	21.7	17.3	24.7
Once per day	3.1	5.4	0.0	3.3	7.8	10.4	18.2	21.9
5–6 times per week	0.0	0.0	0.0	3.3	0.8	3.5	3.6	5.5
3–4 times per week	0.0	0.0	0.0	5.0	3.1	10.4	3.6	8.2
1–2 times per week	3.1	0.0	3.6	18.3	10.9	3.5	3.6	4.1
1–3 times per month	3.1	8.1	3.6	6.7	17.2	7.0	8.2	6.8
Rarely/Never	90.6	86.5	89.1	55.0	46.9	39.1	41.8	27.4
**Males** Application frequency	0–4 years	5–8 years	9–12 years	13–17 years	18–42 years	43–52 years	53–65 years	66+ years
Four times or more per day	0.0	0.0	0.0	0.0	0.0	0.0	0.0	0.0
2–3 times per day	2.7	0.0	2.4	0.0	1.4	1.3	0.0	0.0
Once per day	0.0	0.0	0.0	2.1	2.8	0.0	0.0	1.7
5–6 times per week	0.0	0.0	0.0	0.0	0.0	0.0	0.0	0.0
3–4 times per week	0.0	0.0	0.0	2.1	1.4	0.0	0.0	0.0
1–2 times per week	2.7	0.0	0.0	2.1	0.0	1.3	1.3	0.0
1–3 times per month	0.0	1.8	0.0	2.1	0.0	2.6	0.0	0.0
Rarely/Never	94.6	98.2	97.6	91.5	94.4	94.8	98.8	98.3

Table S13. PCP co-use patterns for adult males.

**PCP combination**	**no. of users**	**% users [a]**
sunscreen, face cream, body lotion, aftershave, hand cream, lip care	33	13.5
sunscreen, face cream, body lotion, aftershave, hand cream	26	10.6
sunscreen, aftershave, hand cream	16	6.5
sunscreen, face cream, aftershave, hand cream, lip care	15	6.1
sunscreen, face cream, hand cream	12	4.9
sunscreen, aftershave, hand cream, lip care	11	4.5
sunscreen, hand cream	10	4.1
sunscreen, face cream, body lotion, hand cream	10	4.1
sunscreen, body lotion, aftershave, hand cream	8	3.3
sunscreen, face cream, body lotion, hand cream, lip care	8	3.3
sunscreen, aftershave	6	2.4
aftershave, hand cream	6	2.4

Table S13. *Cont.*

sunscreen, face cream, aftershave, hand cream	6	2.4
sunscreen, face cream	5	2.0
sunscreen, hand cream, lip care	5	2.0
sunscreen, face cream, hand cream, lip care	5	2.0
sunscreen, lip care	4	1.6
sunscreen, face cream, aftershave	4	1.6
sunscreen, face cream, body lotion, aftershave	4	1.6
face cream, hand cream	3	1.2
sunscreen, body lotion, lip care	3	1.2
face cream, aftershave, hand cream	3	1.2
aftershave, hand cream, lip care	3	1.2
sunscreen, body lotion, hand cream, lip care	3	1.2
face cream, body lotion, aftershave, hand cream	3	1.2
sunscreen, body lotion	2	0.8
body lotion, aftershave	2	0.8
sunscreen, body lotion, aftershave	2	0.8
sunscreen, body lotion, hand cream	2	0.8
sunscreen, body lotion, aftershave, hand cream, lip care	2	0.8
sunscreen, face cream, body lotion, aftershave, hand cream, lipstick	2	0.8
face cream, aftershave	1	0.4
body lotion, hand cream	1	0.4
hand cream, lip care	1	0.4
sunscreen, face cream, body lotion	1	0.4
sunscreen, face cream, lip care	1	0.4
sunscreen, aftershave, lip care	1	0.4
face cream, hand cream, lip care	1	0.4
body lotion, aftershave, hand cream	1	0.4
sunscreen, face cream, body lotion, lip care	1	0.4
sunscreen, face cream, aftershave, lip care	1	0.4
sunscreen, body lotion, aftershave, lip care	1	0.4
face cream, aftershave, hand cream, lip care	1	0.4
sunscreen, face cream, body lotion, aftershave, lip care	1	0.4
sunscreen, face cream, aftershave, hand cream, lipstick	1	0.4
sunscreen, aftershave, hand cream, lip care, lipstick	1	0.4
face cream, body lotion, aftershave, hand cream, lip care	1	0.4
sunscreen, face cream, body lotion, hand cream, lip care, lipstick	1	0.4
sunscreen, face cream, aftershave, hand cream, lip care, lipstick	1	0.4
sunscreen, face cream, body lotion, aftershave, hand cream, makeup, lip care	1	0.4
sunscreen, face cream, body lotion, aftershave, hand cream, lip care, lipstick	1	0.4
sunscreen, face cream, body lotion, aftershave, hand cream, makeup, lip care, lipstick	1	0.4

[a] Overall 245 adult male respondents reported product co-use.

Table S14. PCP co-use patterns for adult females.

PCP combination	no. users	% users [a]
sunscreen, face cream, body lotion, hand cream, makeup, lip care, lipstick	71	24.1
sunscreen, face cream, body lotion, hand cream, lip care	37	12.5
sunscreen, face cream, body lotion, hand cream, lip care, lipstick	31	10.5
sunscreen, face cream, body lotion, hand cream, makeup, lip care	27	9.2
sunscreen, face cream, body lotion, aftershave, hand cream, makeup, lip care, lipstick	14	4.7
sunscreen, face cream, body lotion, hand cream	13	4.4
sunscreen, face cream, body lotion, hand cream, lipstick	10	3.4
sunscreen, face cream, hand cream, lip care	7	2.4
sunscreen, face cream, body lotion, aftershave, hand cream, lip care	7	2.4
sunscreen, face cream, body lotion, hand cream, makeup, lipstick	7	2.4
sunscreen, face cream, body lotion, aftershave, hand cream, lip care, lipstick	7	2.4
sunscreen, hand cream	4	1.4
sunscreen, face cream, body lotion, aftershave, hand cream, makeup, lip care	4	1.4
sunscreen, face cream, body lotion	3	1.0
face cream, body lotion, hand cream, lip care	3	1.0
sunscreen, face cream, hand cream, lip care, lipstick	3	1.0
sunscreen, face cream, body lotion, aftershave, hand cream, lipstick	3	1.0
sunscreen, face cream	2	0.7
sunscreen, face cream, hand cream	2	0.7
sunscreen, body lotion, hand cream	2	0.7
face cream, hand cream, makeup, lip care	2	0.7
sunscreen, face cream, body lotion, hand cream, makeup	2	0.7
sunscreen, face cream, body lotion, lip care, lipstick	2	0.7
sunscreen, face cream, hand cream, makeup, lip care	2	0.7
sunscreen, face cream, hand cream, makeup, lipstick	2	0.7
sunscreen, face cream, body lotion, makeup, lip care, lipstick	2	0.7
sunscreen, face cream, hand cream, makeup, lip care, lipstick	2	0.7
sunscreen, lip care	1	0.3
face cream, hand cream	1	0.3
aftershave, hand cream	1	0.3
sunscreen, face cream, lip care	1	0.3
sunscreen, hand cream, lip care	1	0.3
face cream, body lotion, hand cream	1	0.3
sunscreen, face cream, body lotion, lip care	1	0.3
sunscreen, face cream, body lotion, lipstick	1	0.3
sunscreen, face cream, hand cream, makeup	1	0.3
sunscreen, face cream, hand cream, lipstick	1	0.3
sunscreen, face cream, makeup, lip care	1	0.3
sunscreen, face cream, lip care, lipstick	1	0.3
sunscreen, body lotion, hand cream, lip care	1	0.3
sunscreen, face cream, body lotion, aftershave, hand cream	1	0.3

Table S14. *Cont.*

sunscreen, face cream, aftershave, hand cream, lipstick	1	0.3
sunscreen, body lotion, hand cream, makeup, lip care	1	0.3
face cream, body lotion, aftershave, hand cream, lip care	1	0.3
face cream, body lotion, hand cream, makeup, lip care	1	0.3
face cream, body lotion, hand cream, lip care, lipstick	1	0.3
sunscreen, face cream, body lotion, aftershave, hand cream, makeup	1	0.3
sunscreen, face cream, aftershave, hand cream, makeup, lipstick	1	0.3
face cream, body lotion, hand cream, makeup, lip care, lipstick	1	0.3
sunscreen, face cream, body lotion, aftershave, hand cream, makeup, lipstick	1	0.3
sunscreen, body lotion, aftershave, hand cream, makeup, lip care, lipstick	1	0.3

[a] Overall 295 adult female respondents reported product co-use.

Table S15. PCP co-use patterns for children and adolescent males.

**PCP combination**	**no. users**	**% users [a]**
sunscreen, face cream, body lotion, lip care	33	21.2
sunscreen, face cream, body lotion	18	11.5
sunscreen, lip care	16	10.3
sunscreen, face cream, body lotion, hand cream, lip care	16	10.3
sunscreen, body lotion, lip care	13	8.3
sunscreen, hand cream, lip care	10	6.4
sunscreen, face cream, hand cream, lip care	9	5.8
sunscreen, body lotion, hand cream, lip care	6	3.8
sunscreen, face cream	6	3.8
sunscreen, body lotion	5	3.2
sunscreen, face cream, lip care	4	2.6
sunscreen, hand cream	3	1.9
sunscreen, face cream, body lotion, hand cream	3	1.9
sunscreen, face cream, hand cream	2	1.3
sunscreen, face cream, body lotion, hand cream, makeup, lip care	2	1.3
sunscreen, face cream, body lotion, aftershave, hand cream, makeup, lip care, lipstick	2	1.3
sunscreen, lipstick	1	0.6
sunscreen, face cream, aftershave	1	0.6
sunscreen, face cream, body lotion, aftershave	1	0.6
sunscreen, face cream, aftershave, hand cream	1	0.6
sunscreen, face cream, aftershave, lip care	1	0.6
sunscreen, body lotion, aftershave, lip care	1	0.6
sunscreen, aftershave, hand cream, lip care	1	0.6
sunscreen, face cream, body lotion, hand cream, lip care, lipstick	1	0.6

[a] Overall 156 children and adolescent male respondents reported product co-use.

Table S16. PCP co-use patterns for children and adolescent females.

PCP combination	no. users	% users
sunscreen, face cream, body lotion, hand cream, lip care	29	18.4
sunscreen, face cream, body lotion	17	10.8
sunscreen, face cream, body lotion, lip care	17	10.8
sunscreen, face cream, body lotion, hand cream, makeup, lip care, lipstick	10	6.3
sunscreen, body lotion, lip care	8	5.1
sunscreen, body lotion	7	4.4
sunscreen, face cream, body lotion, hand cream, makeup, lip care	7	4.4
sunscreen, body lotion, hand cream, lip care	6	3.8
sunscreen, face cream, body lotion, hand cream, lip care, lipstick	6	3.8
sunscreen, face cream, body lotion, hand cream	5	3.2
sunscreen, face cream, hand cream, lip care	5	3.2
sunscreen, face cream	4	2.5
sunscreen, face cream, body lotion, lip care, lipstick	4	2.5
sunscreen, lip care	3	1.9
sunscreen, hand cream, lip care	3	1.9
sunscreen, face cream, body lotion, aftershave, hand cream, lip care	3	1.9
sunscreen, face cream, body lotion, aftershave, hand cream, makeup, lip care, lipstick	2	1.3
sunscreen, face cream, hand cream	2	1.3
sunscreen, face cream, lip care	2	1.3
sunscreen, body lotion, hand cream	2	1.3
sunscreen, body lotion, hand cream, lip care, lipstick	2	1.3
sunscreen, face cream, body lotion, aftershave, hand cream, makeup, lip care	2	1.3
sunscreen, face cream, aftershave	1	0.6
sunscreen, face cream, lipstick	1	0.6
sunscreen, body lotion, lipstick	1	0.6
sunscreen, body lotion, hand cream, lipstick	1	0.6
sunscreen, face cream, body lotion, aftershave, hand cream	1	0.6
sunscreen, face cream, body lotion, aftershave, lip care	1	0.6
sunscreen, body lotion, aftershave, hand cream, lip care	1	0.6
sunscreen, body lotion, hand cream, makeup, lip care	1	0.6
sunscreen, hand cream, makeup, lip care, lipstick	1	0.6
sunscreen, face cream, body lotion, makeup, lip care, lipstick	1	0.6
sunscreen, face cream, hand cream, makeup, lip care, lipstick	1	0.6
sunscreen, face cream, body lotion, aftershave, hand cream, lip care, lipstick	1	0.6

[a] Overall 158 children and adolescent female respondents reported product co-use.

Figure S1. Correlation between use frequencies of the eight different PCP categories by gender and life-stage subgroup.

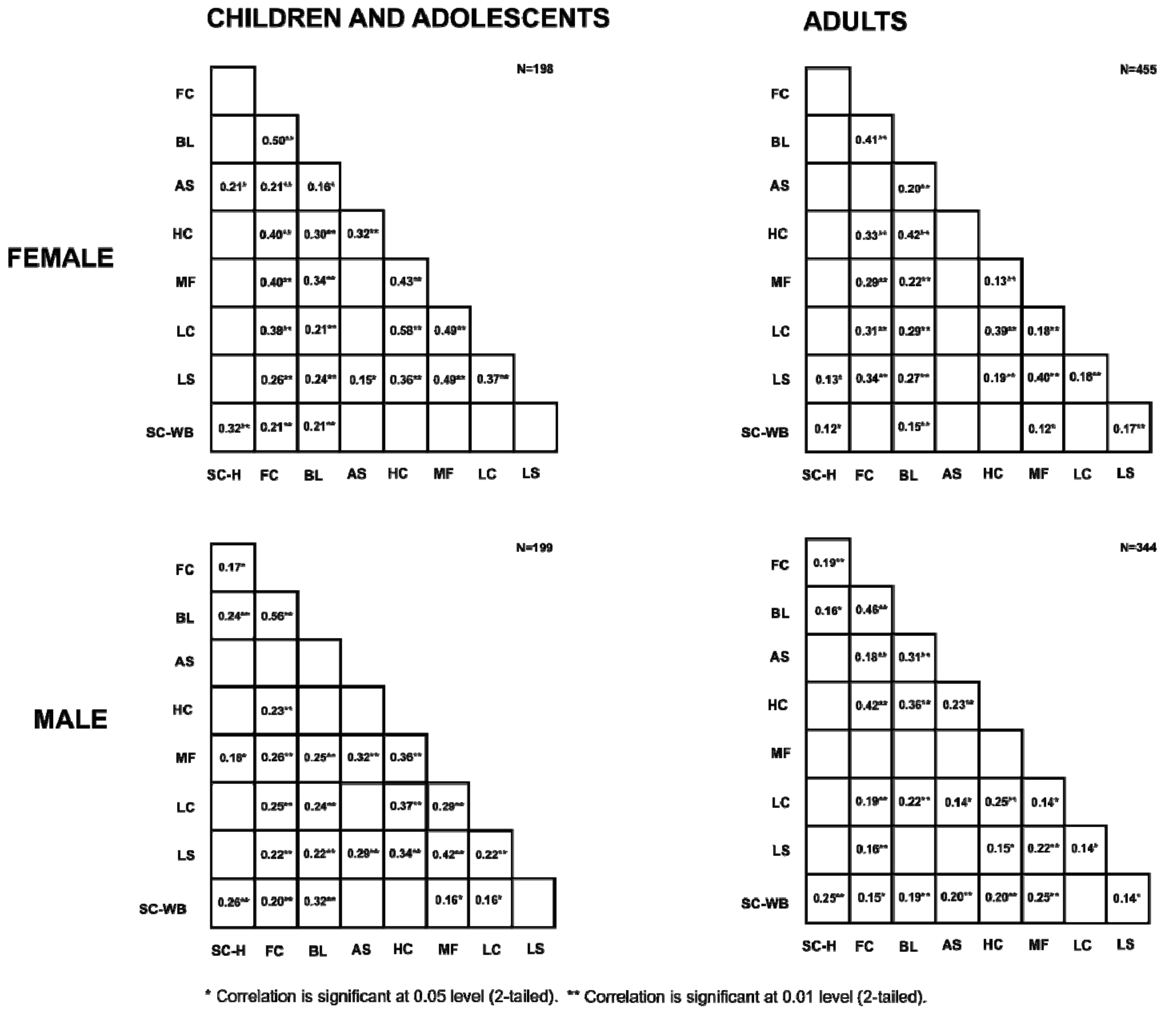

Zero use included; FC (face cream); BL (body lotion); AS (aftershave); HC (hand cream); MF (makeup foundation); LS (lipstick); LC (lip care); SC-WH (sunscreen whole body application in summer); SC-H (sunscreen head only application in winter).

© 2013 by the authors; licensee MDPI, Basel, Switzerland. This article is an open access article distributed under the terms and conditions of the Creative Commons Attribution license (http://creativecommons.org/licenses/by/3.0/).
